# Supplementary material for: Pooled PPIseq: Screening the SARS-CoV-2 and human interface with a scalable multiplexed protein-protein interaction assay platform
Source: PLoS One. 2025 Jan 17;20(1):e0299440. doi: 10.1371/journal.pone.0299440 (PMC11741623; doi:10.1371/journal.pone.0299440)
Supplement: S1 File — (HTML) [file pone.0299440.s001.html]

Yeast transformation, based Gietz


# Yeast transformation, based Gietz

Darach Miller

2023-10-31

# Purpose

To get DNA into yeast, specifically tuned for getting a non-replicating vector into the nucleus for integration using a Cre-lox induction via Gal.

You should get more than 100k with 100ng of an episomal plasmid.

# Concept

This is mostly from Gietz, as well as inspired by Kevin Roy’s disciplined adherence and guidance to adhere to the classic Gietz method. I just changed the scale and added the Base+Dex step! Not much.

The theory is that the procedure disrupts the cell wall structure while precipitating DNA all over and amongst it, such that at least some transforming DNA gets up against the membrane. Then upon relief of the heat+LiAc and replenishing with glucose, this triggers some sort of endocytic event that internalizes the precipitated DNA.

There are ideas that fork off from this that have not been tested.

# Solutions to prepare

## ssDNA

Prepare as in Gietz’s method, the 2mg/mL solution. Kevin Roy made a bunch, so we use that.

## PEG, LiAc as in Gietz

Carefully prepare 50% PEG 33501 by dissolving in water with stir bar and with a bit of heat, then pouring and rinsing this beaker into a graduated cylinder, and QSing to volume where that’s 50% w/v. Either vacuum filter or autoclave 15min.

Also prepare 1M LiAc as in Gietz, autoclave 15min.

## Growth and recovery media

### 20% glucose

mix with stir bar, autoclave

### 20% galactose

filter, keep cold

### 2x YPD

This is YPD … but 2x. So 4% glucose.

### Base+Dex, Base+Gal

Use a 4x YNB stock, dilute with water and carbon source to ~2%.

# Protocol

1. Part 1
   1. *Start overnight of yeast*, from either plate or from frozen stock2. Use a pipette to get a bit of frozen glycerol/culture and put it in YPD (1x or 2x is fine). Let this grow to saturation, so this will take >16 hours, best to start these around noon for the next day depending on how much you innoculate.
2. Part 2
   1. *Measure the OD* of a ~1/200 dilution, blanked against empty media diluted to the same amount.
   2. Based on this, *back dilute this overnight* into 50mL of 2x YPD per transformation reaction, and put at 30C shaking. 3 Aim for about 0.15 OD, but do not overshoot this. You can grow longer, not shorter time4.
   3. Let grow for at least 4 hours. *Monitor OD of 1/4 dilutions of the culture*, blanked against a 1/4 dilution of 2xYPD in water. 5
      - During this time, prepare 10x of the TRAFO multimix per 50mL of culture sampled, *sans* ssDNA, and leave at RT:

        | what | uL in 1x mix |
        | --- | --- |
        | 50% PEG 3350 | 240 |
        | 1M LiAc | 36 |
        | water + DNAs | 34 |
        | ssDNA 2mg/mL, freshly denatured | (50) |
      - Leave the ssDNA on ice, which should be freshly denatured (95C 10min then to ice immediately) or thawed from being previously freshly denatured (can freeze thaw about three times at least from being denatured).
3. Part 3
   1. *When the culture hits 0.8 OD* (and do try to hit this spot on), split the 2x YPD culture into 50mL conicals, *spin to collect* (3000g 5min here on out).
   2. Dump super. Knock tubes to loosen the pellet, then *add approximately 25mL of sterile water* and shake to resuspend.
   3. Spin again.
   4. Dump super. Knock tubes to loosen the pellet, then *add approximately 10mL of sterile water* and shake to resuspend. 6.
   5. Spin again.
      1. During this step, add the ssDNA to the multimix, and vortex to mix well. Leave at RT.
   6. Dump super, quick spin pulse, *pipette all supernatant water out*.
   7. Knock tubes to loosen pellet, and *add 10x TRAFO multimix*. Resuspend with pipette and vortexing, vigorously mix it completely. Tap it to try and get most liquid into the bottom.
   8. *Put into 42C water bath for 60min*. At the start, swirl about every 30s for a minute or two to let it equilabrate, then swirl at 20min and 40min.
4. Part 4
   1. *At 60min, spin 3000g 5min*.
   2. *Pipette off the supernatant* 7, quick spin, and get pretty much all off it off. Do not wash!
   3. Tap to loosen pellet, then *add 10mL (for a 10x here) of Base+Dex* recovery media, invert, shake, and vortex a bit to resuspend and wash the inside of the tube with it.
   4. Put at *30C shaking 2 hours*.
5. Part 5
   1. Spin, dump supernatant, quick spin and pipette off the remaining.
   2. Resuspend with about 5mL of Base, Base+Gal, or water to *wash the pellet/tube of leftover glucose*. Spin again, and dump/spin/aspirate all.
   3. Loosen pellet and *resuspend with 10mL Base+Gal*. Count cell density on a hemacytometer.
   4. Put at 30C shaking overnight, in a shake flask or two culture tubes.
6. Part 6
   1. Count cell density to see if it expanded at all. Likely not.
   2. Spin to collect cells, resuspend in about 1mL total volume with Base+Dex (unsure if this is any different than water at this stage).
   3. Plate 100uL on plates, with ~10 beads to spread even and dryly. Grow to select on these plates.

---

1. 3350 is a lot easier to handle than 8000, so this may yield better transformation due to accuracy, maybe↩
2. Different people have different philosophies about starting culture. I subscribe to the notion that bottlenecking is Bad -school, and so will isolate my new strain from a clone, grow it up, verify it, and then start my cultures from that frozen stock directly as much as possible. Again, people do things differently, it’ll usually be just the same.↩
3. It is best to add too little, then directly measure the OD of this mixture again, then adjust up conservatively.↩
4. unless you’re diluting from an exponential pre-culture↩
5. According to a 2008 master’s thesis from Gietz’s lab, the transformation efficiency goes up, then down, then back up at 4 hours, so don’t short this! Do the 4 hours, at least. This is probably due to cell cycle changes, so don’t muck with it - without controls.↩
6. At this stage, each 1mL has ~1x of cells ready for TRAFO multimix, so if you’re doing a large scale then just leave them in the conical and treat as a 10x reaction↩
7. You could save if the transforming DNA is precious, I’ve not tried re-using it but would be suprised if you can’t get anything from that. Maybe add more ssDNA, maybe not.↩
