# Supplementary material for: Pooled PPIseq: Screening the SARS-CoV-2 and human interface with a scalable multiplexed protein-protein interaction assay platform
Source: PLoS One. 2025 Jan 17;20(1):e0299440. doi: 10.1371/journal.pone.0299440 (PMC11741623; doi:10.1371/journal.pone.0299440)
Supplement: S2 File — (HTML) [file pone.0299440.s002.html]

Yeast library-scale DNA extraction


# Yeast library-scale DNA extraction

Darach Miller

2023-10-31

# Purpose

Get genomic DNA out of yeast for barseq, clean enough for ~1ug/50uL rxn PCR.

# Concept

The aim is to (1) lyse and disrupt cells to then permit (2) fractionating any DNA using CTAB, PVP, salt, chloroform, and an alcohol precipitation. To lyse in a less genotype/condition/growth-rate biased way we use a thorough bead beating, but this is correlated with the production of some Mystery Contaminant

Notes: - Katja Schwartz was working on this idea too, did it first with columns, but Darach doesn’t trust columns at that time (does now), so silica beads idea is from Guillaume Diss - Inspired by plant CTAB protocols for the CTAB+Pvp, but I don’t believe these are actually necessary. Maybe. I dunno. - Cells lysed by beads, \*ases mostly stopped by salt and EDTA, RNaseA is for degrading RNA into littler pieces. Heat and chloroform helps denature and separate some macro molecules, CTAB helps dissolve membrane and sequester polysaccharides (theoretically) and PVP helps with polyphenols (theoretically). High salt keeps CTAB going into the chloroform or soluble fraciton, theoretically.

Properties of the Mystery Contaminant:

- makes solution post alcohol precip kinda thick, slightly hazy
- contributes to high absorbance in 260nm (~10-15x of qubit-estimate of DNA)
- recalcitrant to RNAseA treatment
- loves to precipitate in alcohol, precipitates with DNA in alcohol precipitations, using a few different salts
- precipitates in PEG along with DNA
- precipitates onto silica from the qiaex2 kit, but is not eluted as much at 50C *as it is at 65C*
- is not readily precipitated by 0.3x etOH 1-2M NaCl precip as recommended by PacBio *but something else is precipitating!*
- *might* be precipitated with the sodium perchlorate in the QX1 buffer?

Current speculation is that the Mystery Contaminant is:

- polyphenols covalently bound to the DNA
- RNA somehow made recalcitrant to RNAseA
- some sort of other molecules that looks like nucleic acid, but is like a polysaccharide or something
- cell wall components, maybe that stick to nucleic acids?

# Solutions to prepare

## Xbuffer (extraction buffer)

Result should be a clear buffer, a little viscous, with some bubbles.

1. Put in a 50mL conical ~30mL clean (DI or better) water
2. Add to that:
   - 1g PVP
   - 1g CTAB
   - 4.09g NaCl
   - 5mL 1M Tris-Hcl (~7.5pH tested)
   - 1mL 0.5mM EDTA
3. Invert/shake to dissolve, using 65C incubation for ~10min to help. It should completely dissolve in this within the hour, if it’s not stuck on the bottom as a pellet.
4. QS to 50mL final volume using water (about 10mL volume left to add).

# Protocol steps

## Anticipated collection of cells

1. Centrifuge 50mL of yeast cells1, about 5min at about 3000 rcf.
2. Dump supernatant out as spent media.
3. Options:

   1. In 50mLs
      1. Spin again briefly.
      2. Aspirate all media from the pellet, without largely disturbing/losing pellet.
      3. Tap 50mL conical tube on it’s side sharply on the bench to loosen the pellet to make it easier to resuspend later, and freeze it at -20C. This takes up a lot of space
   2. In eppendorfs (not tested but suggested that you test this!)

      1. Vortex the pellet up with the remaining media, and use that to transfer to eppendorf.
      2. Spin that as hard as you’d like, >8krcf is good.
      3. Aspirate all remaining media, maybe knock the tube about (rattle rasp on the tube racks is handy) to loosen the pellet to save time on extracts, then freeze it at -20C.

## Extraction

1. Part 1 - bead beating
   1. From library sample pellets in tubes, thaw these out for a bit. This is not tested, but just get them a bit loosened back up, liquid.
   2. As or after they’ve thawed, add 400uL Xbuffer and vortex to resuspend in this, and use this to transfer this onto a ~2mL bead tube with ~0.3mL glass beads and an o-ring screw top.
   3. Add ~20ug of RNAseA2 (1uL of 20mg/mL Monarch NEB), invert to mix, and run in a bead beater3 for 5min.
   4. When beat, move the tube to 65C waterbath for 30min.
2. Part 2 - cook and chloroform
   1. Take out tube and add 400uL 24:1 chloroform:isoamyl alcohol4.
   2. Invert and vortex to mix, then put back at 65C waterbath for 15min.
3. Part 3 - separate and precip
   1. Spin the tube 2min max speed RT.
   2. Aspirate the top layer, the aqueous supernatant5 into a new tube.
   3. Add ~600uL 24:1 chloroform:isoamyl alcohol, and vortex to mix.
   4. Spin 2min max speed RT.
   5. Aspirate supernatant to new tube, careful to not take any interphase.
   6. To this clear yellow supernatant, add 0.7x volumes isopropanol6, invert/shake a few times to mix, then spin 2min max speed RT.
   7. Note the large pellet. Aspirate the supernatant and discard, and wash the pellet by adding 1mL of 70% etOH and inverting the tube7.
   8. Spin to make sure pellet is down, then aspirate supernatant and discard it. Spin again, and aspirate with a smaller pipette, then dry the tube/pellet for 10min.
4. Part 4 - resuspend and clean
   1. Add 200uL of TE to the pellet. Resuspend.
      1. One way to do this is to incubate on bench ~10min to loosen it, and use a pipette to complete resuspend it8.
      2. The other is to incubate on bench ~30min, with periodic vortexing at approximately 10 and 20minutes. Then it should resuspend by  
         vortexing alone.
   2. Using the Qiaex2 kit, add 1 volume (200uL) of QX1 buffer, and vortex briefly to mix. Note if there’s cloudy precipitate or not.
   3. Spin the tubes (without adding qiaex2 solution!!!) for 30s at max speed on a bench centrifuge ~21krcf.
   4. Aspirate the supernatant away from a tiny gray pellet or bead at the the bottom of the tube (the precipitate from the previous previous step.
   5. To this QX1 yellow supernatant, then add 30uL of qiaex2 solution (this is volume for a ~2.5e9 cells input, adjust as needed), vortex to mix.
   6. Over the course of ~10min incubation at RT, vortex every 2min.
   7. Spin 30s 16krcf, and aspirate supernatant.
   8. Resuspend beads (Qiaex2 pellet) with 0.5mL PE to wash, then spin again. Pellet is very thick at this point, you can dig in and mix with the tip and it should come back off.
   9. Aspirate supernatant. Use 0.5mL PE to wash (resuspension optional), invert tubes to wash tube, spin again.
   10. Aspirate supernatant. Spin, aspirate again, spin, aspirate, then let dry 10-15min.
   11. Resuspend beads with 30uL of EB buffer, completely. Put at 50C9 for >10min.
   12. Spin to collect beads, aspirate supernatant to new tube.
   13. Resuspend beads with 30uL of EB buffer, completely. Put at 50C for >10min.
   14. Spin to collect beads, aspirate supernatant to that same new tube.
5. Part 5 - quantify
   1. Quantify 1uL with a qubit BR dsDNA kit, and the nanodrop. Yield should be >10ug in the total 60uL, hopefully >15ug, but qubit. Nanodrop should read about 2-4x the estimate by absorbance. 260/280 should be ~1.9 and 260/230 should be ~1.8 to 2.3 or so.

---

1. Anticipating that you’re doing ~2-5e7 cells per mL in SD add-back media with a lab strain of Saccharomyces like 4741. So this is being optimized/tested on ~2.5e9 cell pellet.↩
2. You can multimix this in with a batch of the Xbuffer, and just add that. It’s 1uL RNAseA per extraction.↩
3. We use a Biospec 607.↩
4. This will pop some bubbles, so you can “waterfall” it to multiple samples↩
5. Note the separation, clear yellow supernatant, thick interphase with most everything there, then clean bottom↩
6. You may have about 380uL at this point.↩
7. Or, you can add and remove with the same pipette to save a step, this seems to work.↩
8. The suspension will be slightly slightly hazy, slightly viscous, and bubbly.↩
9. The kit recommends 50C for longer DNA, so we do that. I tried 65C once and it got lots of mystery contaminant, ie high 260nm and cloudiness.↩
