# Supplementary material for: Pooled PPIseq: Screening the SARS-CoV-2 and human interface with a scalable multiplexed protein-protein interaction assay platform
Source: PLoS One. 2025 Jan 17;20(1):e0299440. doi: 10.1371/journal.pone.0299440 (PMC11741623; doi:10.1371/journal.pone.0299440)
Supplement: S3 File — (HTML) [file pone.0299440.s003.html]

Yeast library-scale amplicon prep for sequencing, mjtF and evoR2 primers


# Yeast library-scale amplicon prep for sequencing, mjtF and evoR2 primers

Darach Miller

2023-10-31

# Purpose

Amplify DNA product for illumina sequencing, from a lot of complex gDNA.

# Concept

Too much gDNA, especially with contaminants, will inhibit PCR. So we try to use the cheap polymerase for several reactions to generate a smaller amplicon from that, using the UMI primers, then we purify that away from primers and genomic DNA using Ampure beads.

# Solutions to prepare

### 20% PEG 8000 2.5M NaCl

Prepare, either combining 40% PEG and 5M NaCl stocks, or making it up to volume for dissolving the PEG 8000 using NaCl stock diluted.

# Multiplexing, considerations thereof

Use combinations of inside-outside primers, but keep each end double-unique. So that means use combos like [ A11A, A22A, B11B, … ] but not combos like [ A11A, A12B, B12B, … ] .

Then each set of double-unique ends can be used to filter out chimeras, and the combinations of the inside-outside primers (numbers and letters above) can be used to give exponential increase in multiplexing samples per primer sets. Neato!

# Input

You need clean-enough genomic DNA. The associated protocol with the bead-beating and CTAB/PVP/chloroform/Qiaex2 (silica beads) extraction works well enough routinely.

This was tested and seems to work fine with 2ug per 50uL inputs, so I chose to use 1ug for routine dependable PCRs.

# Protocol

1. Part 1 - R1 reaction
   1. Thaw reagents (except for polymerase of course) and setup multimix:

      | reagent | 1x | 9x | 22x |
      | --- | --- | --- | --- |
      | 1ug of clean-enough gDNA | ? | ? | ? |
      | water | QS | QS | QS |
      | 5x OneTaq GC buffer | 10ul | 90uL | 220uL |
      | 25mM magnesium chloride | 0.8ul | 7.2uL | 17.6uL |
      | 10mM (each) dNTPs | 1ul | 9uL | 22uL |
      | MjtF primer, 10uM | 2.5uL | 22.5uL | 55uL |
      | EvoR2 primer, 10uM | 2.5uL | 22.5uL | 55uL |
      | OneTaq DNA polymerase | 0.25uL | 2.25uL | 5.5uL |
   2. Dispense 50uL of 1x reactions into single PCR tubes1, either as one multimix to a multimix plus genomic DNA template.
   3. Put onto pre-warmed T100 thermocycler (lid at 105C) and run program:

      - 94C 4min
      - 3 cycles of:
        - 94C 1min
        - 52C 30s
        - 68C 1min
      - 4C hold
2. Part 2 - pool reactions and precipitate
   1. Pool all reactions for one sample into one tube, eppendorf or falcon as needed.
   2. Add 1/10th volume of 3M sodium acetate solution, then 0.7x volumes of isopropanol.
   3. Spin, at least 10min RT for eppendorfs, and of unknown duration for the falcons (??????)
   4. Observe that there is a good pellet, and remove supernatant.
   5. Add 80% etOH to wash the pellet and tubes, and spin again at high speed for at least 30s.
   6. Remove supernatant, spin again, remove all supernatant.
   7. Let this dry about 10 minutes.
3. Part 3 - bead work
   1. Resuspend pellet with 25uL2 of water.
   2. Add 2x volumes of Ampure XP beads (pre-warmed to RT), mix with pipette at least 10 strokes.
   3. Incubate RT 10min, then mix again.
   4. Collect beads on magnet, and allow to fractionate (may take ~2-5min).
   5. Aspirate supernatant, consider saving it.
   6. Wash bead pellet and tube with ~0.5mL 80% etOH, so pipette down the tube on the dispense. Wait at least 30s, then pipette it back out and off.
   7. Wash bead pellet and tube with ~0.5mL 80% etOH, so pipette down the tube on the dispense. Wait at least 30s, then pipette it back out and off.
   8. Quick spin the tube in a microfuge, put back on beads, and aspirate all liquid from the tube.
   9. Immediately resuspend the beads in 20uL water, and mix. Incubate 5min.
   10. Add 11uL (0.55x volumes) of 20% PEG 8000 2.5M NaCl solution, mix well, incubate 10min RT.
   11. Mix, and separate beads on the magnet.
   12. Aspirate the supernatant to a new tube *(this is your library!)*
   13. Add 77.5uL (2.5x volumes) of 100% etOH to this library supernatant, put on ice 10min, then spin 10min RT max speed.
   14. Aspirate supernatant, and wash pellet with ~0.5mL of 80% etOH.
   15. Spin, aspirate, spin, aspirate completely, and let dry 10min on bench.
   16. Resuspend the pellet with 20uL of EB, and then keep on ice.
   17. QC this by measuring 1uL each sample with qubit DNA kit. You should detect a little DNA, but not a lot (or the next PCR will fail).
4. Part 4 - test conditions for the second PCR
   1. Clean your workspace to remove contamination from other libraries, so 1:10 bleach surfaces, and consider separating your PCR and pre-amp work.
   2. Pre-make some qubit HS DNA assay mix, and take the machine so you have quick access.
   3. Setup multimix for the test3 second round of PCR, consider not using separate multiplexing primers, but just using one or a mix of them.

      | reagent | 1x | 3.3x |
      | --- | --- | --- |
      | 1uL of your library from the first step | 1uL | - |
      | water | 12.8uL | 42.24uL |
      | 5x KAPA HiFi GC buffer | 4ul 1 | 3.2uL |
      | 10mM KAPA dNTP mix | 0.6ul | 1.98uL |
      | Second round multiplexing MjtF primer, 10uM | 0.6uL | - |
      | Second round multiplexing EvoR2 primer, 10uM | 0.6uL | - |
      | KAPA HiFi DNA polymerase | 0.40uL | 1.32uL |
   4. Dispense multimix into single PCR tubes, on ice, add 1uL library R1 template that’s been purified away from the gDNA with the beads.
   5. Mix and measure 1uL of the multimix using the HS qubit assay per library, this is your baseline.
   6. Put onto pre-warmed T100 thermocycler (lid at 105C) and run the 19uL test run with program:

      - 95C 1min
      - ??x4
        - 98C 20s
        - 55C 15s
        - 72C 15s
      - 72C hold
   7. At that last hold, pop the tops and take 1uL of the reaction, measure on qubit HS DNA. Quickly decide if you are starting to get amplification off of baseline, and if it’s enough to gel.
   8. Consider running more cycles, if needed, to see anything.
   9. Run 10ul of the reaction out on a gel, 2-3% agarose TAE with ethidium bromide staining, on a real imaging gel box.
5. Part 5 - the second PCR
   1. At this stage, consider the info from your test run, and decide how many cycles you want to amplify your library for. Consider the input ammount, the qubit readings, and how much you want to have.
   2. Clean your workspace to remove contamination from other libraries, so 1:10 bleach surfaces, and consider separating your PCR and pre-amp work.
   3. Once resolved, setup multimix for the second round of PCR5. Be very careful to not be introducing library from the test PCR back into your final PCR.

      | reagent | 1x | 3.3x |
      | --- | --- | --- |
      | half or so of your library from the first step | 10uL | - |
      | water | 24.5uL | 80.85uL |
      | 5x KAPA HiFi GC buffer | 10ul | 33.00uL |
      | 10mM KAPA dNTP mix | 1.5ul | 4.95uL |
      | Second round multiplexing MjtF primer, 10uM | 1.5uL | - |
      | Second round multiplexing EvoR2 primer, 10uM | 1.5uL | - |
      | KAPA HiFi DNA polymerase | 1.0uL | 3.30uL |
   4. Dispense multimix into single PCR tubes, on ice, add 10uL or so library R1 template that’s been purified away from the gDNA with the beads.
   5. Mix and measure 1uL of the multimix using the HS qubit assay per library, this is your baseline.
   6. Put onto pre-warmed T100 thermocycler (lid at 105C) and run the 49uL test run with program:

      - 95C 1min
      - ??x6
        - 98C 20s
        - 55C 15s
        - 72C 15s
      - 4C hold
   7. Purify the library. This should be done on a gel (unless you want to develop and verify that bead cleanup works for getting rid of dimers). So run it out on a 1.2% or so agarose gel, cut the band (the stuff at expected size, and the stuff above it) and clean that up. You may want to use the zymo kit, maybe the qiagen qiaex2 kit. You must decide. But you must purify away the pervasive PCR dimers!

# Cost analysis

I assume you want to do 30ug of input gDNA, so about 2.5e9 copies input, probably 100mL culture (of SD+his+leu+1ug/mL MTX for a PPiSeq library) extracted.

Each R1 is going to be 30 OneTaq reactions at 50uL. Ignoring dNTP or primer costs, this should be ~$8.25 worth of OneTaq (M0480S).

Then, this is pooled and precipitated, and resuspended and purified in one small AmpureXP clean/depletion. 50uL of AmpureXP should cost less than 50 cents.

The final Kapa reaction costs $5 for the 50uL, and < $2 for the 20uL test. So about $7, without primers.

Multiplexing primers are designed to be 60bp, so a full set will cost about $36 of inside/outside first/second. Samples multiplexable will scale as the square of the number of sets you have, so to estimate costs at scale it would be ideally $36 \(\times\sqrt{\text{samples}}\). Roughly.

So the fixed costs are primers, and the per 30ug sample (ie a timepoint) costs are going to be under $20.

---

1. Why singles? Well, that’s how this has been optimized. When someone tests it in tube strips, then we can do that. It most likely works, but it has been known to matter.↩
2. You may want to adjust the volume for different anticipated pellet sizes, DNA inputs. Look up the binding capacity of your beads, use that to guide how much you want to be depleting the gDNA, and go from there.↩
3. Yep, running 20uL as a test to guesstimate how much to amplify. There’s no safe place measure “in flight”, unless you want to snatch 1uL from it during the annealing, and even then you’ll loose water vapor, unless you’re using an oil overlay, which I don’t want to mess with yet.↩
4. You’re going to have to do your own for this. I believe 18x would be informative.↩
5. This is setup to be 20uL for the test, then 1uL for baseline, and ~38uL for the main run. Conditions optimized at 20, assuming it’s still good at ~38uL. Anticipating 3 libraries at a time, because anticipating running 30x reactions per sample in the first round.↩
6. Decide based on the test run.↩
