# Supplementary figures and images for: Pooled PPIseq: Screening the SARS-CoV-2 and human interface with a scalable multiplexed protein-protein interaction assay platform
Source: PLoS One. 2025 Jan 17;20(1):e0299440. doi: 10.1371/journal.pone.0299440 (PMC11741623; doi:10.1371/journal.pone.0299440)

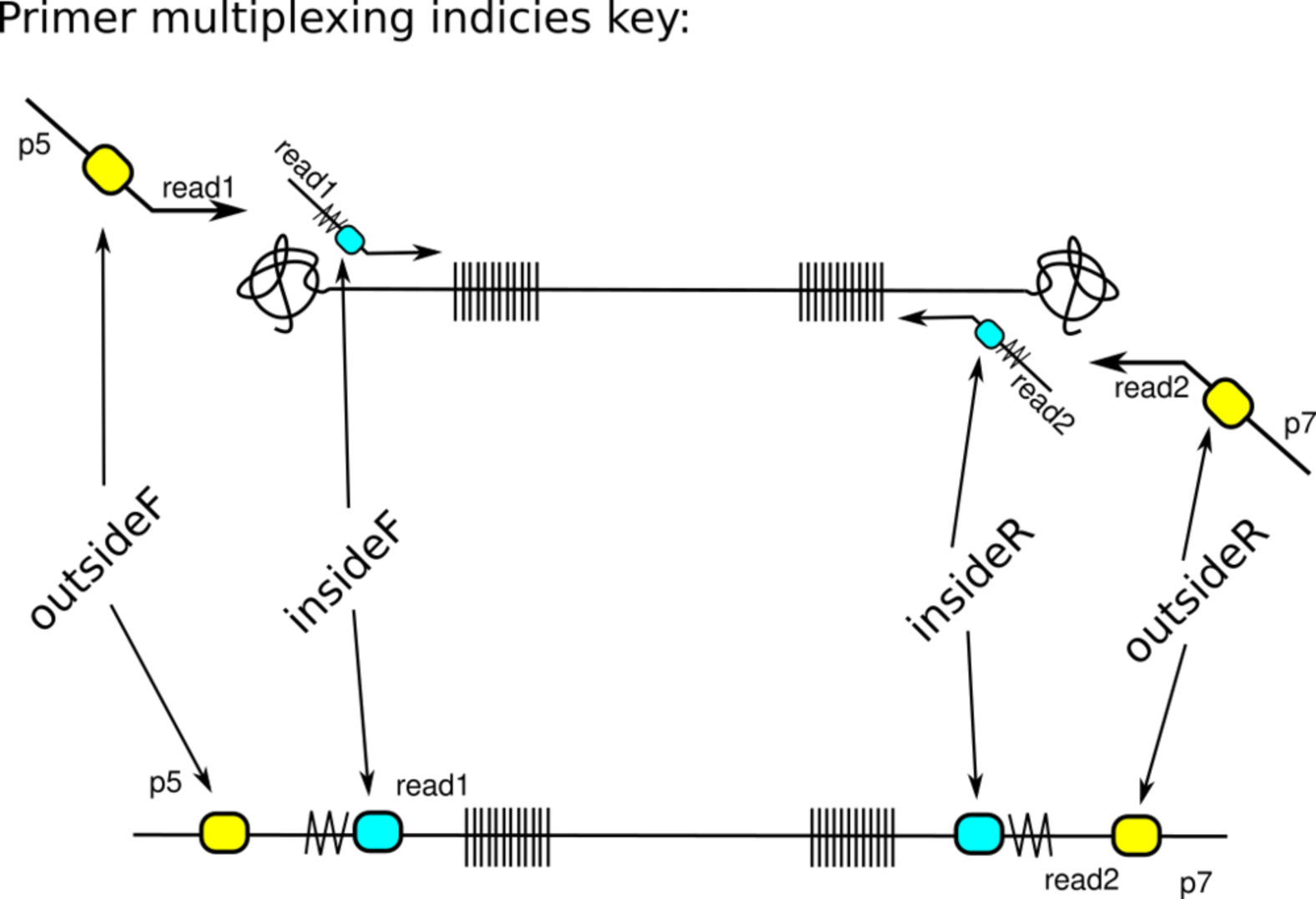

Supplement: S6 File — (TIF) [file pone.0299440.s006.tif]

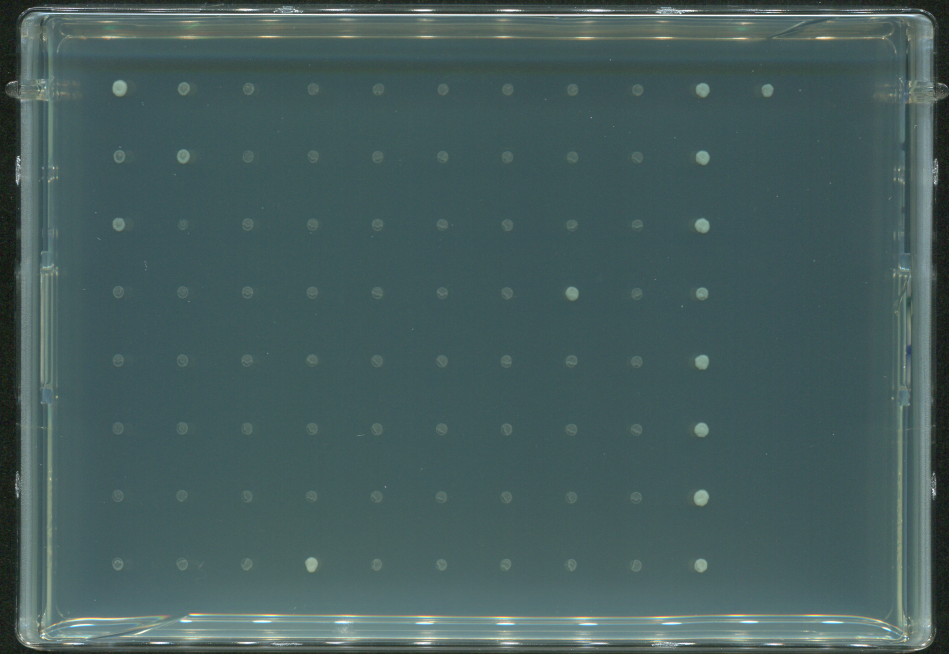

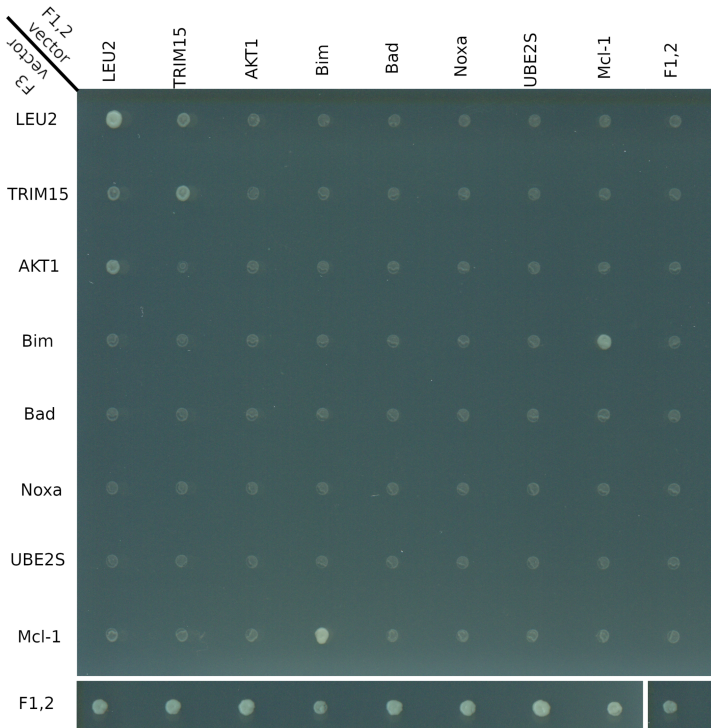

Supplement: S1 Fig — Raw scans of plates used for initial verification that modified plasmids and modified strains still reproduce the mDHFR assay results. Layout as described in S13 Table. (PDF) [file pone.0299440.s007.pdf]

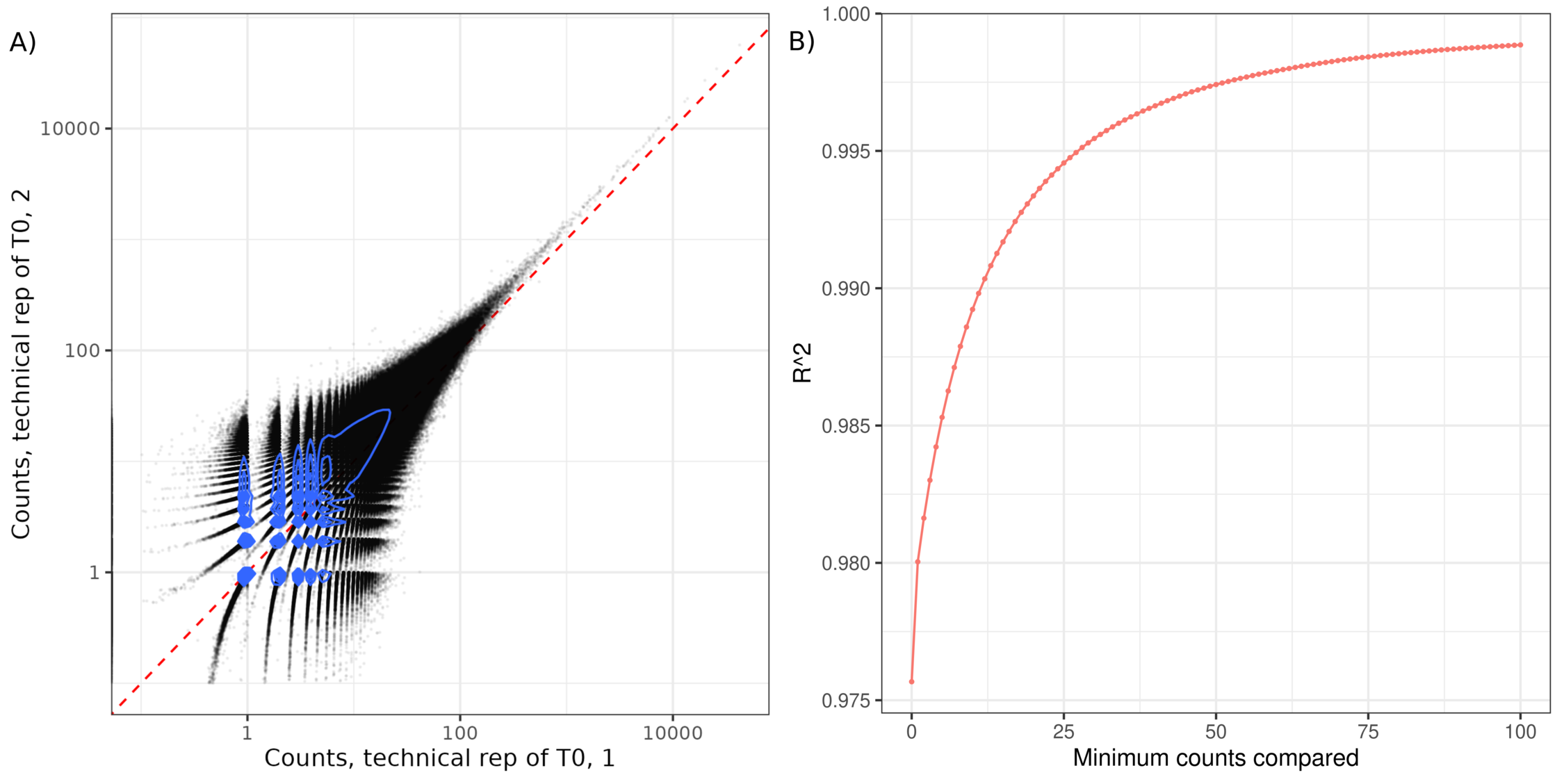

Supplement: S4 Fig — PPIseq double-barcode counts are well correlated. Two biological samples of the initial timepoint’s lineage pool were subject to independent DNA extraction, PCR, and sequencing. A) Chimera-adjusted counts from each sample (Methods). B) R^2 for a subset of the dataset (y-axis), calculated for all observations with both counts greater than the minimum counts threshold (x-axis). (TIF) [file pone.0299440.s010.tif]

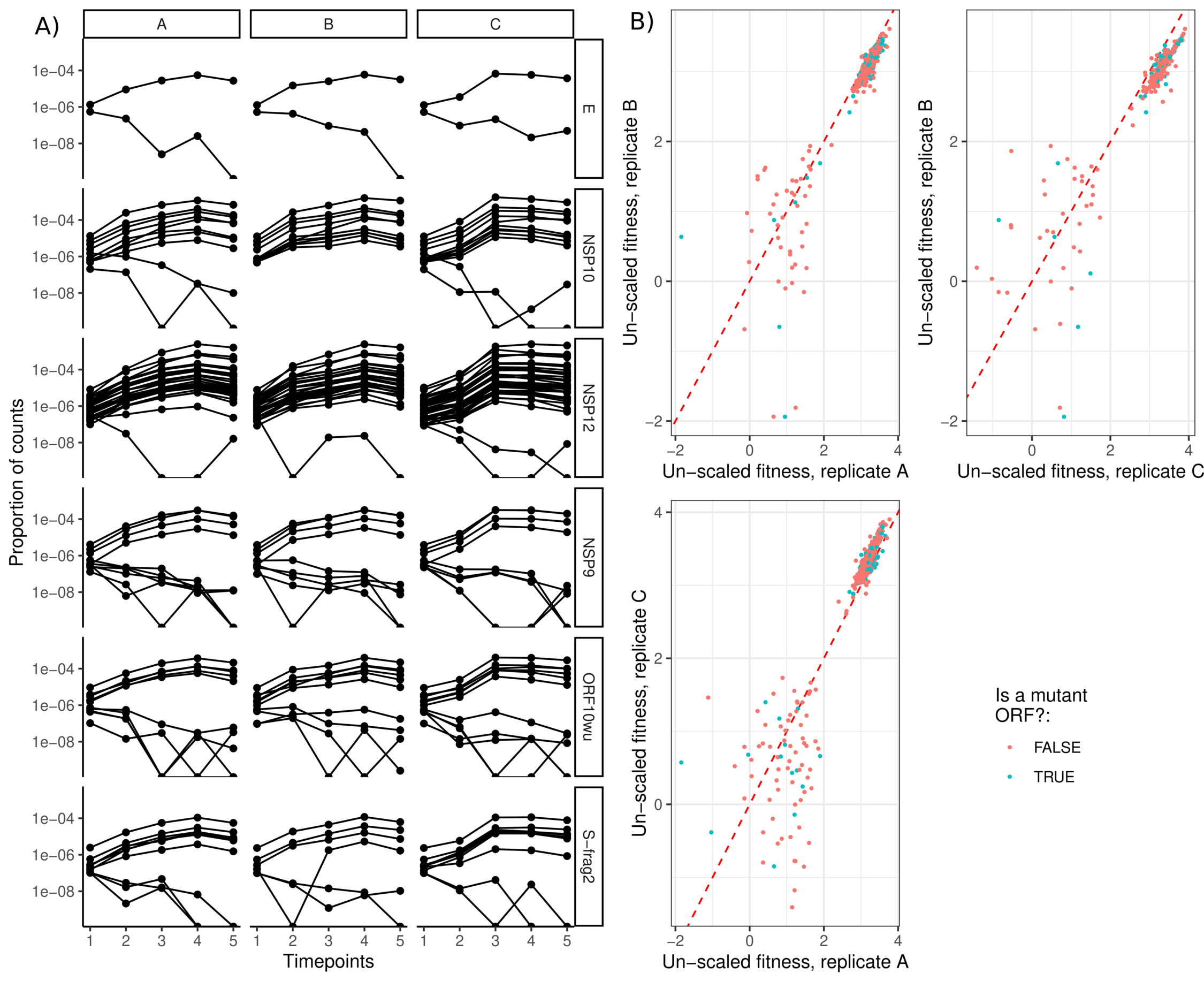

Supplement: S5 Fig — Dropout lineages are consistently less fit and not explained by ORF mutations. A) Examples of lineages for tagged human DHFR and tagged vORF positive controls that are expected to have high fitness. Proportion of counts (y-axis) per lineage is shown for each timepoint (x-axis). Some lineages are not detected in some replicates. B) The un-scaled fitness of each lineage for all tagged human DHFR and tagged vORF positive control lineages are plotted for each replicate (x and y axes). Color indicates if the lineage contains an ORF that was annotated by the long-read plasmid annotation to contain a non-synonymous mutation. (TIF) [file pone.0299440.s011.tif]

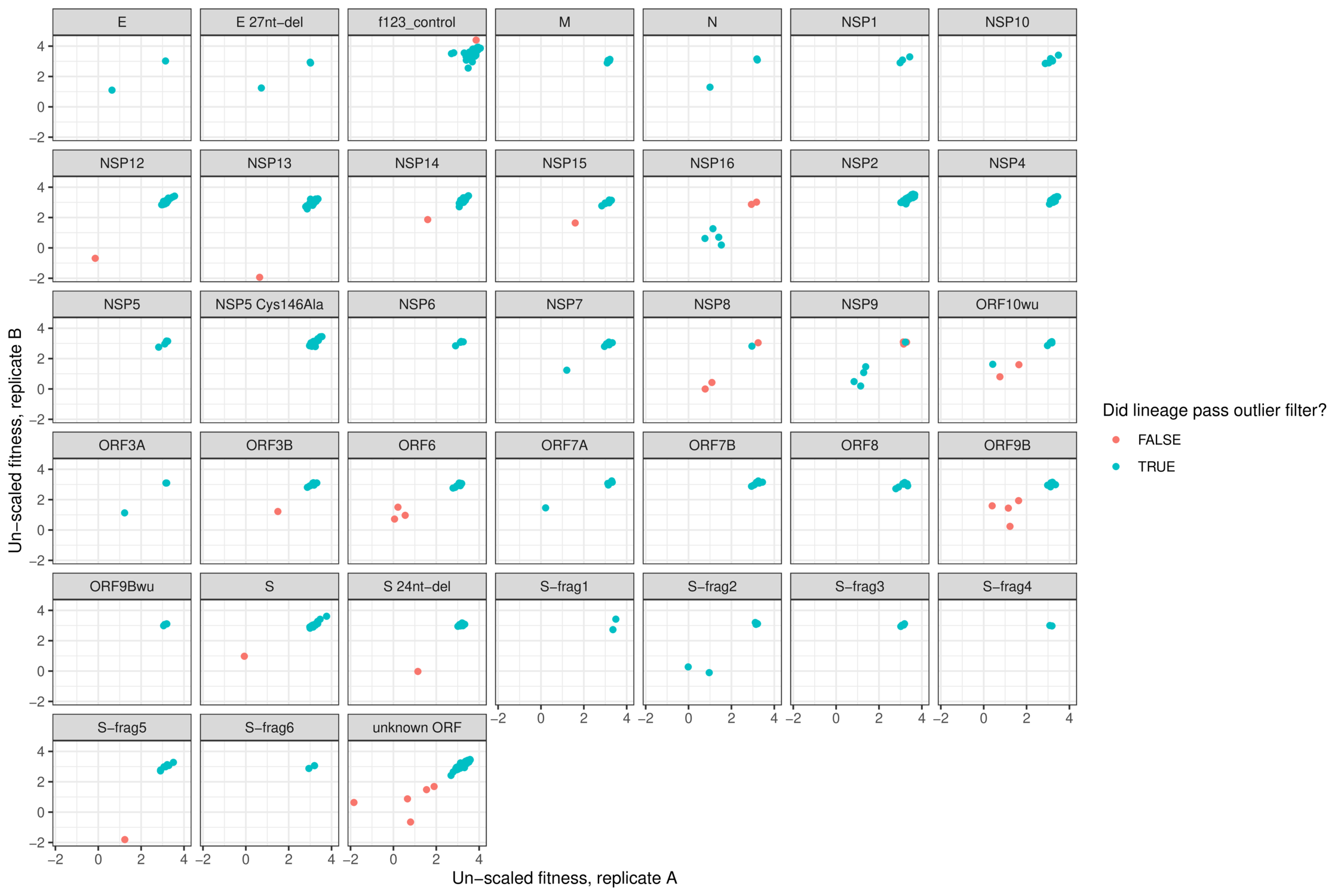

Supplement: S6 Fig — Examples of outlier filtering. For each panel the un-scaled fitness of each lineage for all tagged human DHFR and a certain tagged vORF positive control lineages are plotted, for replicate A (x-axis) against replicate B (y-axis). Color indicates if the lineage passed the outlier filer or not (FALSE indicates exclusion). (TIF) [file pone.0299440.s012.tif]

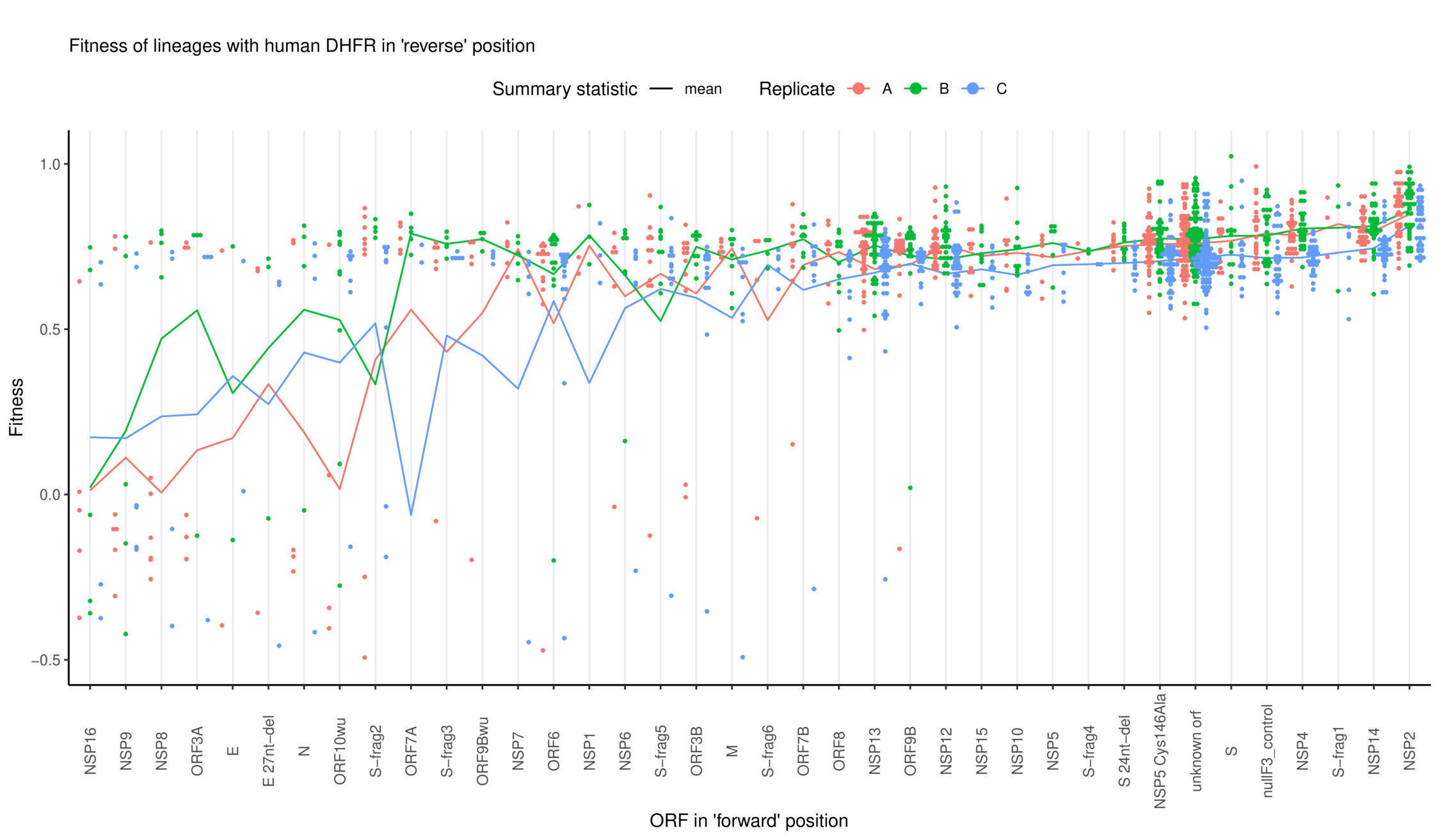

Supplement: S7 Fig — NSP2 tends to have a slight fitness advantage with human DHFR positive control. For each vORF (x-axis) for each replicate (color), the scaled fitness of each lineage of tagged human DHFR and tagged vORF lineages are plotted (y-axis), and the mean of each grouping is indicated by the solid line. (TIF) [file pone.0299440.s013.tif]

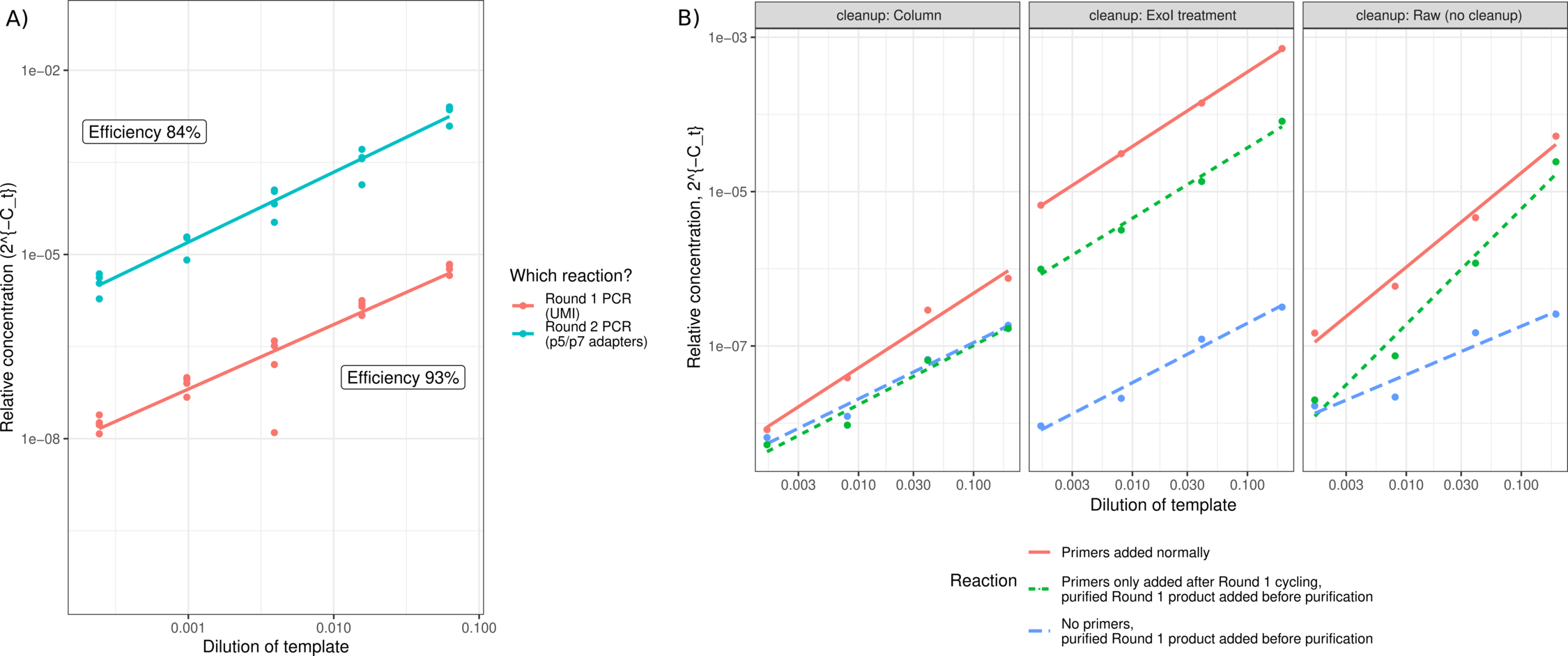

Supplement: S8 Fig — New PPIseq priming sites and PCR protocol are efficient and effective at generating UMI-containing libraries. A) qPCR of Round 1 PCR (genomic DNA template and genomic priming sites) and Round 2 PCR (purified Round 1 template with p5/p7-containing primers) were set up with Kapa HiFi polymerase and SybrGreen dye (Methods). Plotted is the "relative concentration" of the template as estimated from 2^{-C_t} where C_t is the maximum second-derivative cycle threshold calculated by the AB HT7900 qPCR machine. Dilution is from a serial dilution of the template. B) A similar experiment was performed, but the Round 2 PCR was carried out with template either from a normal Round 1 PCR (primers added, thermocycled, then template purified), or from a reaction where the primers were withheld but a purified product of a previous Round 1 template was added, or from a reaction where the primers were withheld until after the cycling but primers and a purified Round 1 template were added just before purification. Three different purification strategies (raw with no purification, column purification, or ExoI treatment) show different efficacy at repressing the primer-after reaction to the same level as the no-primer reaction. (TIF) [file pone.0299440.s014.tif]

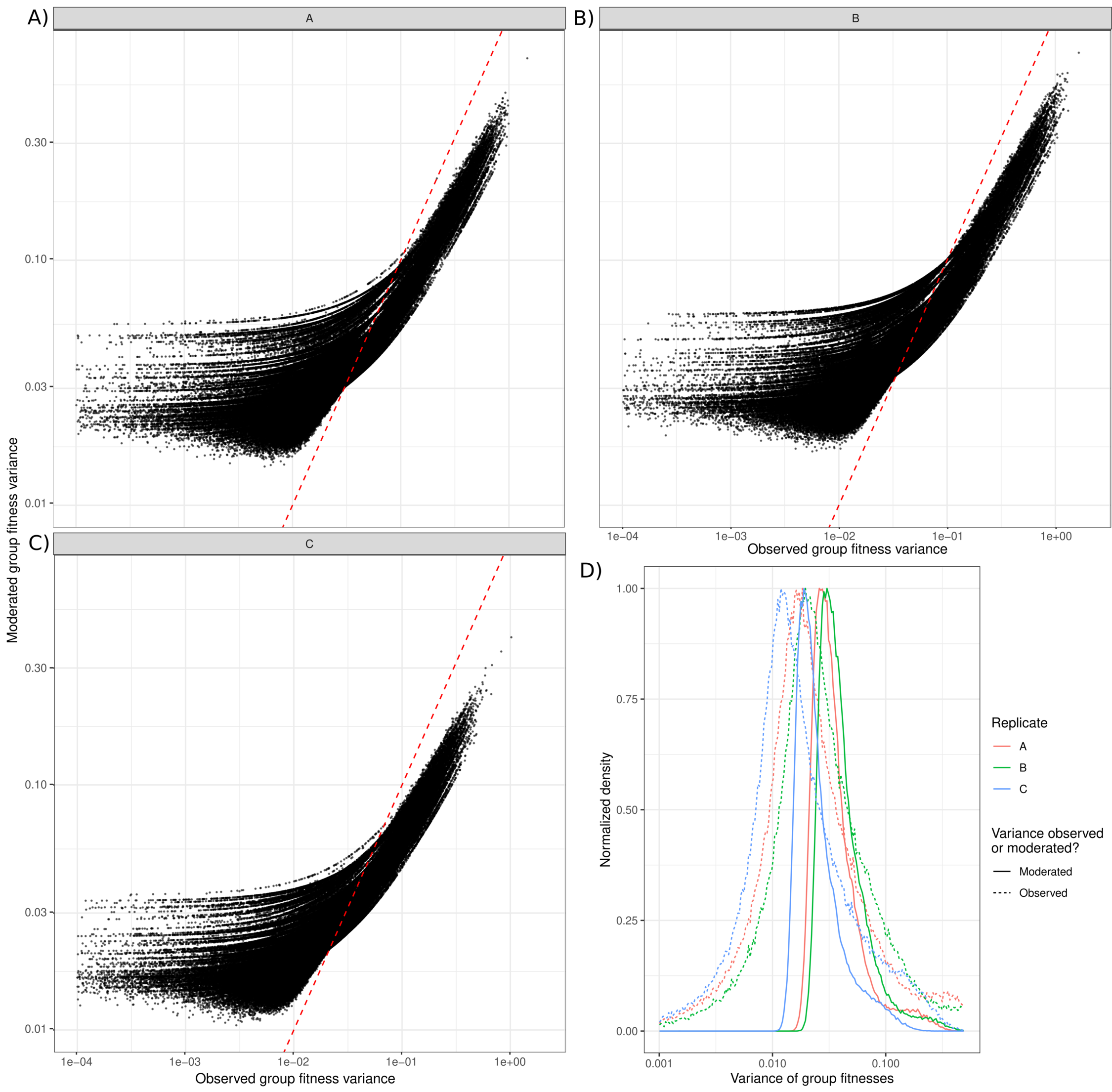

Supplement: S9 Fig — A summary of the variance shrinking approach to moderate false positives. A), B), C) show for each replicate the observed variance of fitnesses within an ORF-ORF lineage group (x-axis) and the moderated, or shrunk group fitness variance used for the one-sided t-test. D) The distribution (y-axis) of the variance in fitnesses per ORF-ORF group (x-axis) is shown for the observed and moderated variance (linetype) for each replicate (color). (TIF) [file pone.0299440.s015.tif]

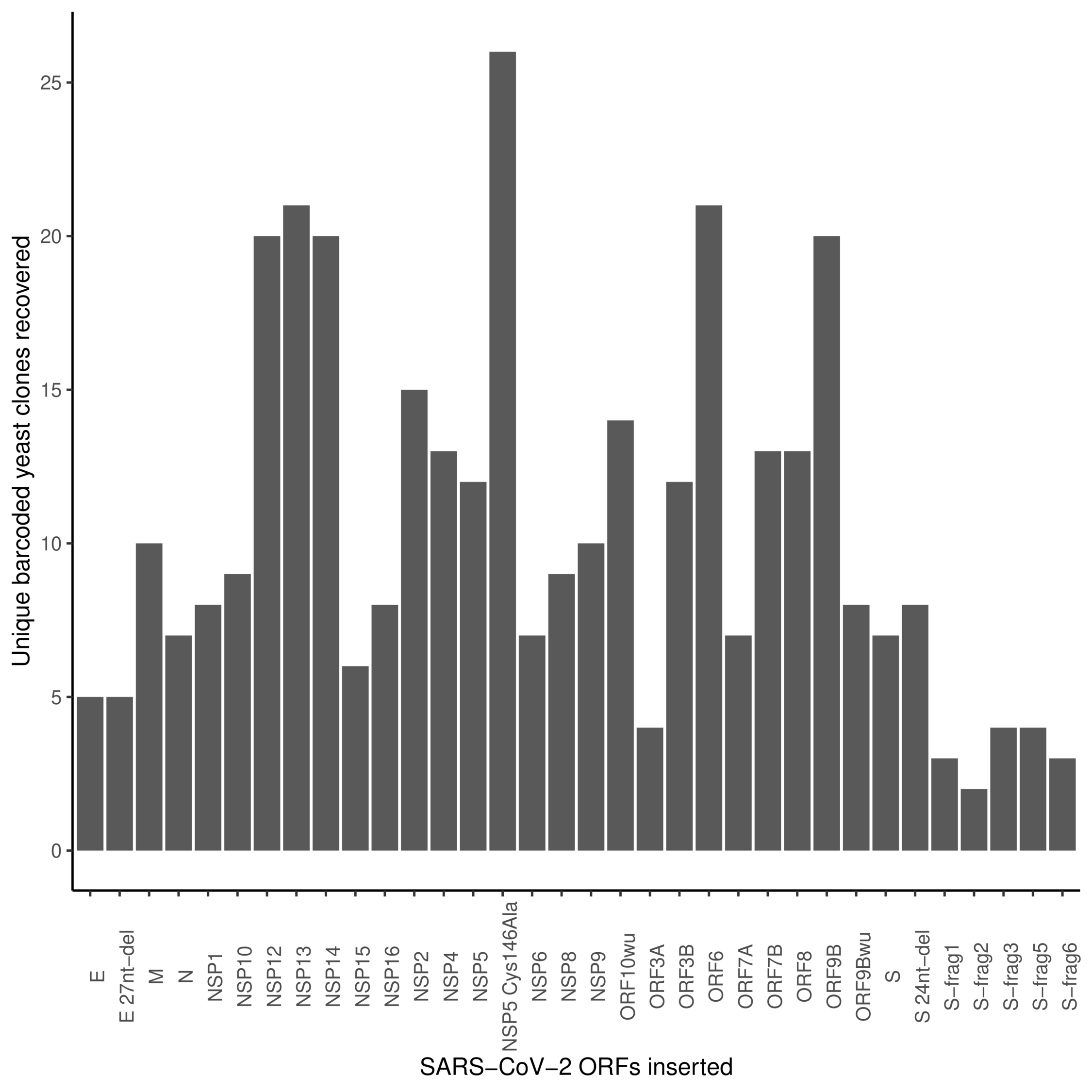

Supplement: S10 Fig — REDIseq is a viable strategy for converting pooled PPIseq haploid precursor yeast strain pools into arrayed libraries. We used dilution to separate pools of PPIseq haploids (yLSL52, yLSL53, yLSL54, yeast that contained integrated F[3]-tagged vORFs) 9 384-well plates. Many wells were left empty by this random dispersion process, but the entire plates were subject to REDIseq wherein these were crossed to plates of compatible iSeq 2.0 strains with known barcodes. For each tagged vORF (x-axis), we recovered some number of uniquely-barcoded haploid clones (y-axis). (TIF) [file pone.0299440.s016.tif]

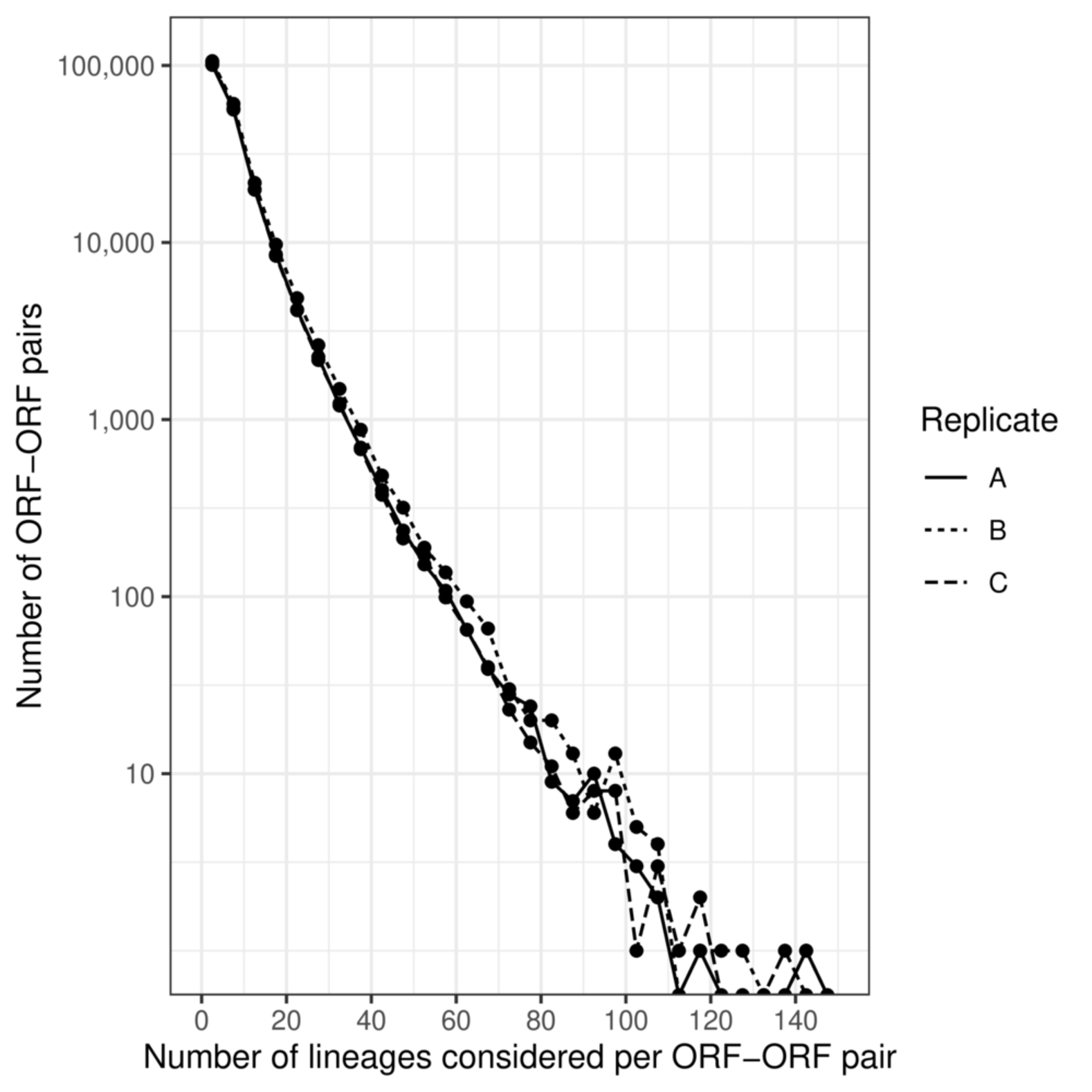

Supplement: S11 Fig — The number of lineages per ORF-ORF combination is well dispersed. The distribution (y-axis) of the number of double-barcoded lineages (x-axis) that assay each ORF-ORF pair is shown. Linetype indicates different replicates. (TIF) [file pone.0299440.s017.tif]
