# Supplementary material for: Pooled PPIseq: Screening the SARS-CoV-2 and human interface with a scalable multiplexed protein-protein interaction assay platform
Source: PLoS One. 2025 Jan 17;20(1):e0299440. doi: 10.1371/journal.pone.0299440 (PMC11741623; doi:10.1371/journal.pone.0299440)
Supplement: S2 Fig — For each timepoint (x-axis), the relative frequency of each lineage (y-axis) is shown for each replicate and each ORF-ORF combination (panel facetting). ’Hit’ or ’non-significant’ label indicates if this data was called as a significant PPI hit, and line color indicates the lineage fitness. (PDF) [file pone.0299440.s008.pdf]

All NULL-NULL negative control lineages. Blue line is a LOESS-smoothed curve, red dashed line is a projected model of the average fitness of all the NULL-NULL lineages with initial abundance of the median abundance of the lineages. This red-dashed line is the same reference point presented on subsequent plots.

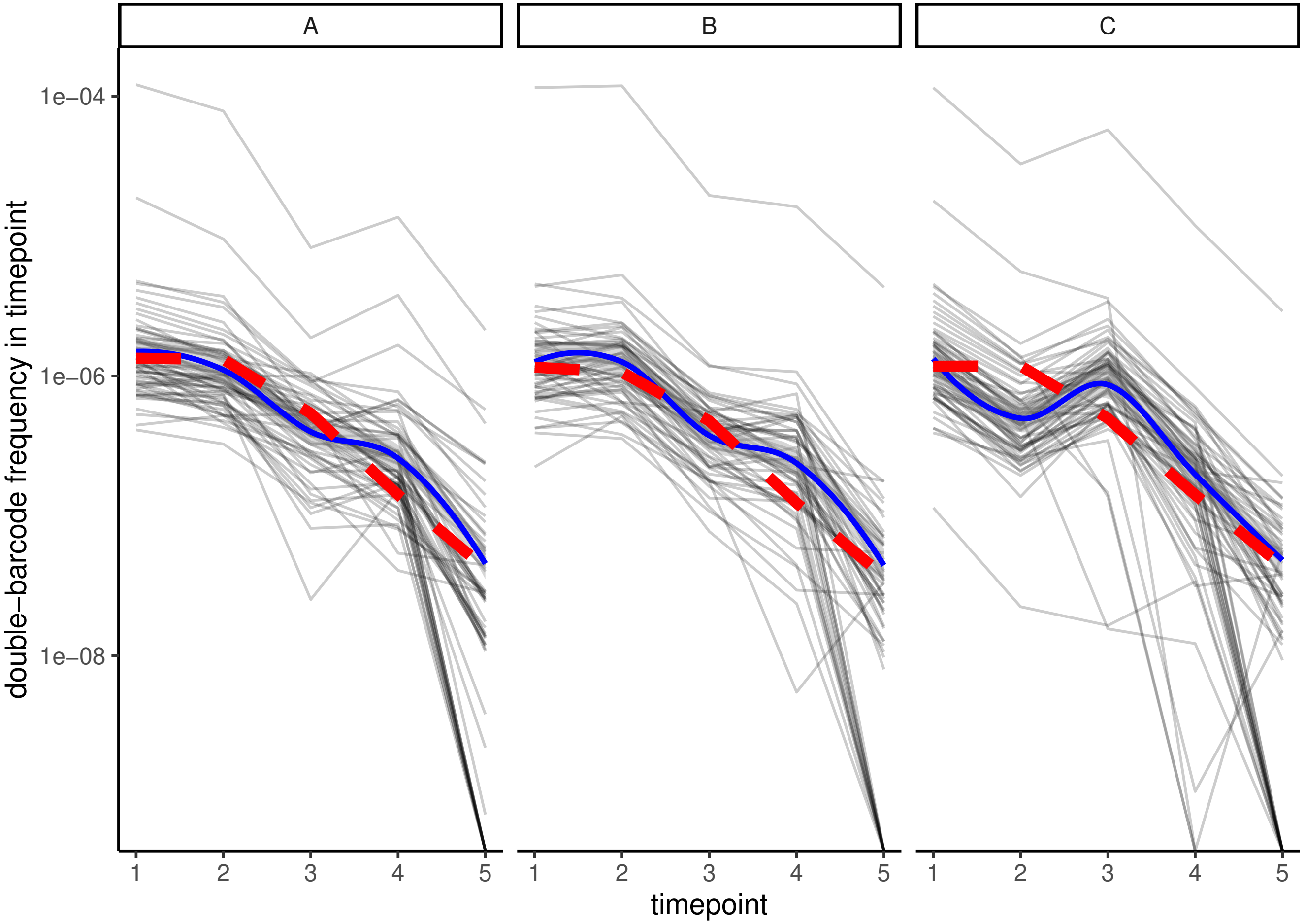

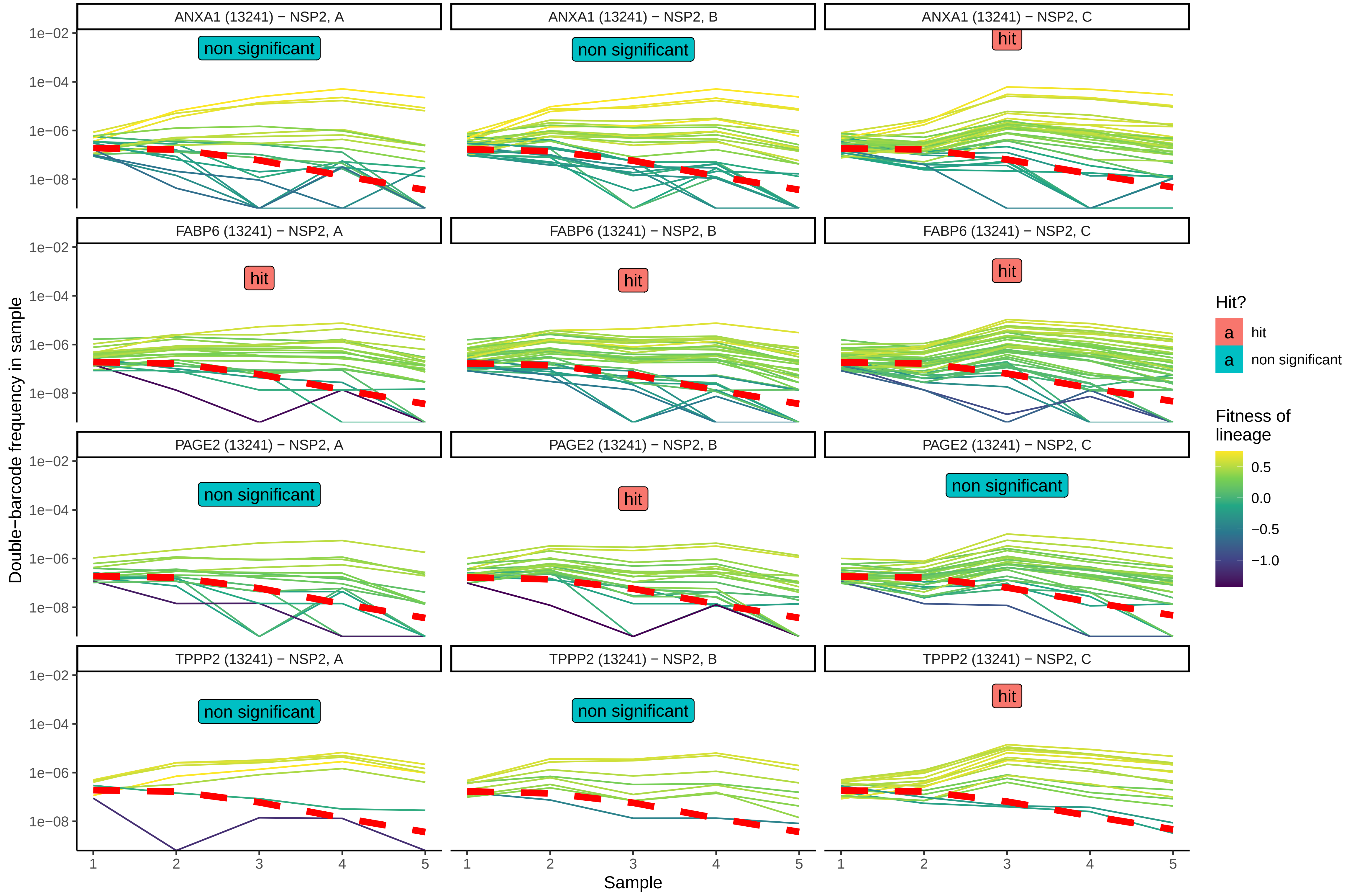
ANXA1 (13241) − NSP2, A
ANXA1 (13241) − NSP2, B
ANXA1 (13241) − NSP2, C
FABP6 (13241) − NSP2, A
FABP6 (13241) − NSP2, B
FABP6 (13241) − NSP2, C
PAGE2 (13241) − NSP2, A
PAGE2 (13241) − NSP2, B
PAGE2 (13241) − NSP2, C
TPPP2 (13241) − NSP2, A
TPPP2 (13241) − NSP2, B
TPPP2 (13241) − NSP2, C
non significant
hit
Double−barcode frequency in sample
Sample
1e−02
1e−04
1e−06
1e−08
1
2
3
4
5
Hit?
hit
non significant
Fitness of lineage
0.5
0.0
−0.5
−1.0

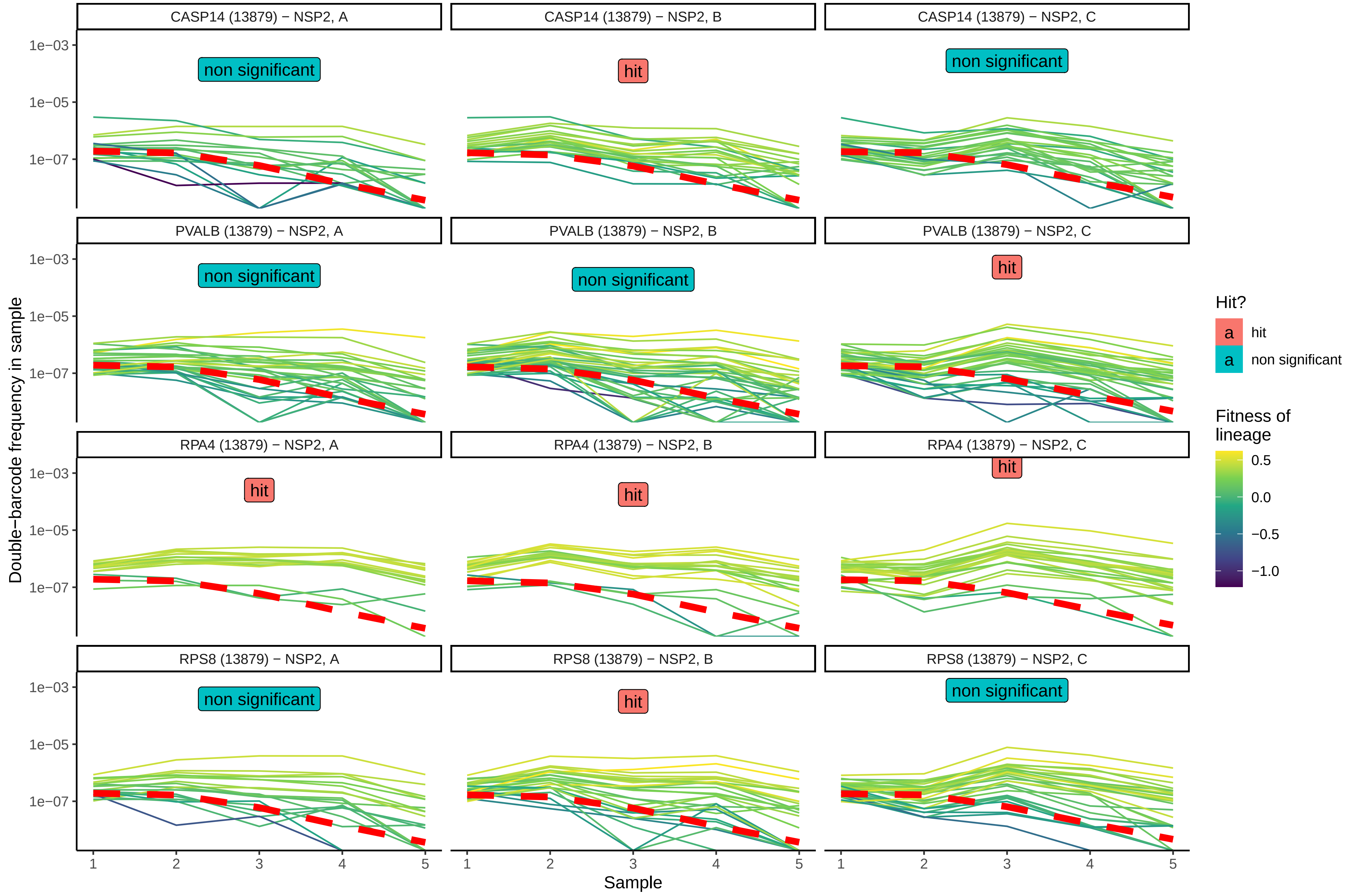

CASP14 (13879) − NSP2, A
CASP14 (13879) − NSP2, B
CASP14 (13879) − NSP2, C
PVALB (13879) − NSP2, A
PVALB (13879) − NSP2, B
PVALB (13879) − NSP2, C
RPA4 (13879) − NSP2, A
RPA4 (13879) − NSP2, B
RPA4 (13879) − NSP2, C
RPS8 (13879) − NSP2, A
RPS8 (13879) − NSP2, B
RPS8 (13879) − NSP2, C
non significant
hit
1e−03
1e−05
1e−07
1
2
3
4
5
Sample
Double−barcode frequency in sample
Hit?
hit
non significant
Fitness of lineage
0.5
0.0
−0.5
−1.0

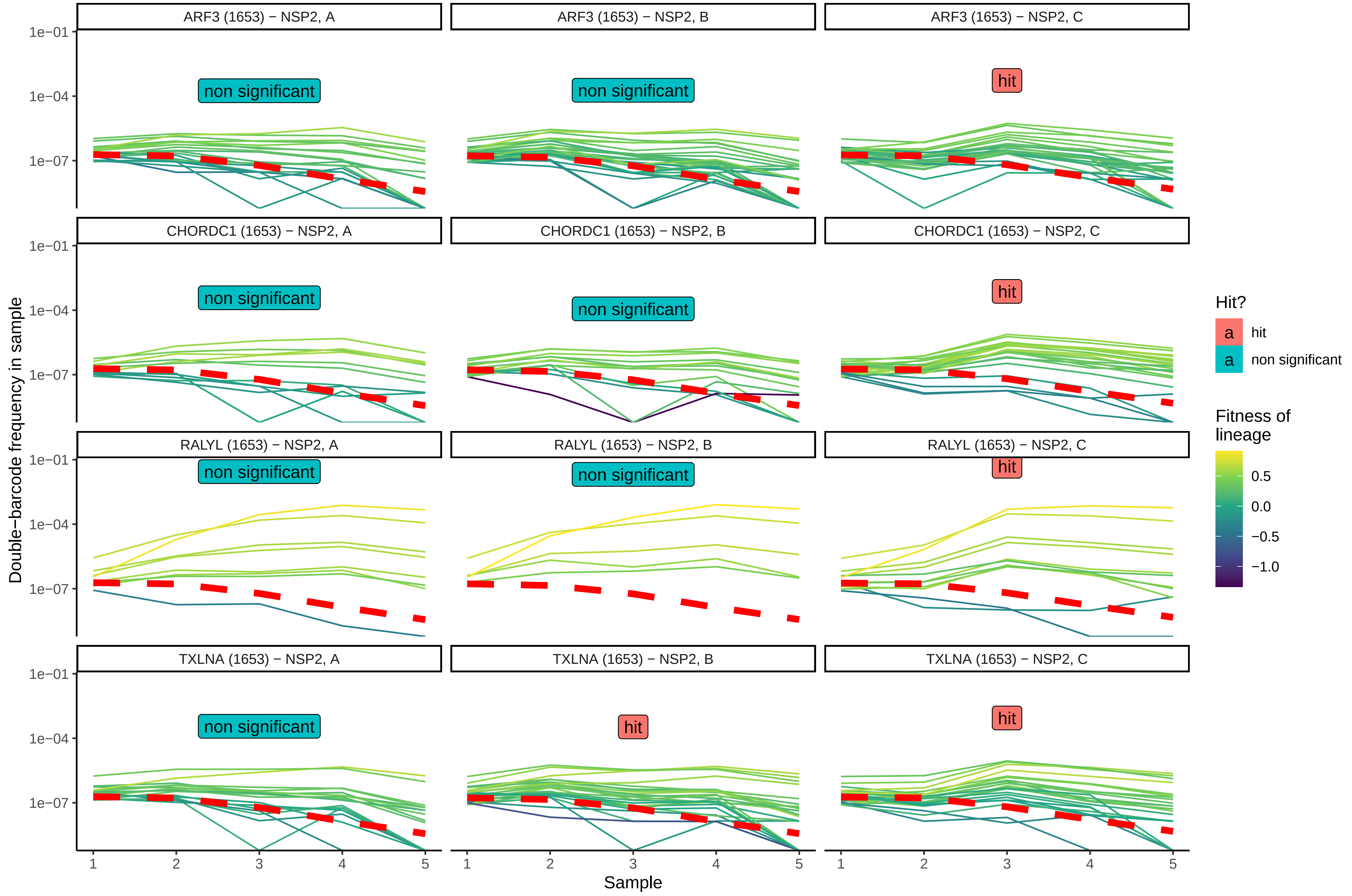

ARF3 (1653) − NSP2, A
ARF3 (1653) − NSP2, B
ARF3 (1653) − NSP2, C
CHORDC1 (1653) − NSP2, A
CHORDC1 (1653) − NSP2, B
CHORDC1 (1653) − NSP2, C
RALYL (1653) − NSP2, A
RALYL (1653) − NSP2, B
RALYL (1653) − NSP2, C
TXLNA (1653) − NSP2, A
TXLNA (1653) − NSP2, B
TXLNA (1653) − NSP2, C
non significant
hit
1e−01
1e−04
1e−07
Double−barcode frequency in sample
Sample
1
2
3
4
5
Hit?
a hit
a non significant
Fitness of lineage
0.5
0.0
−0.5
−1.0

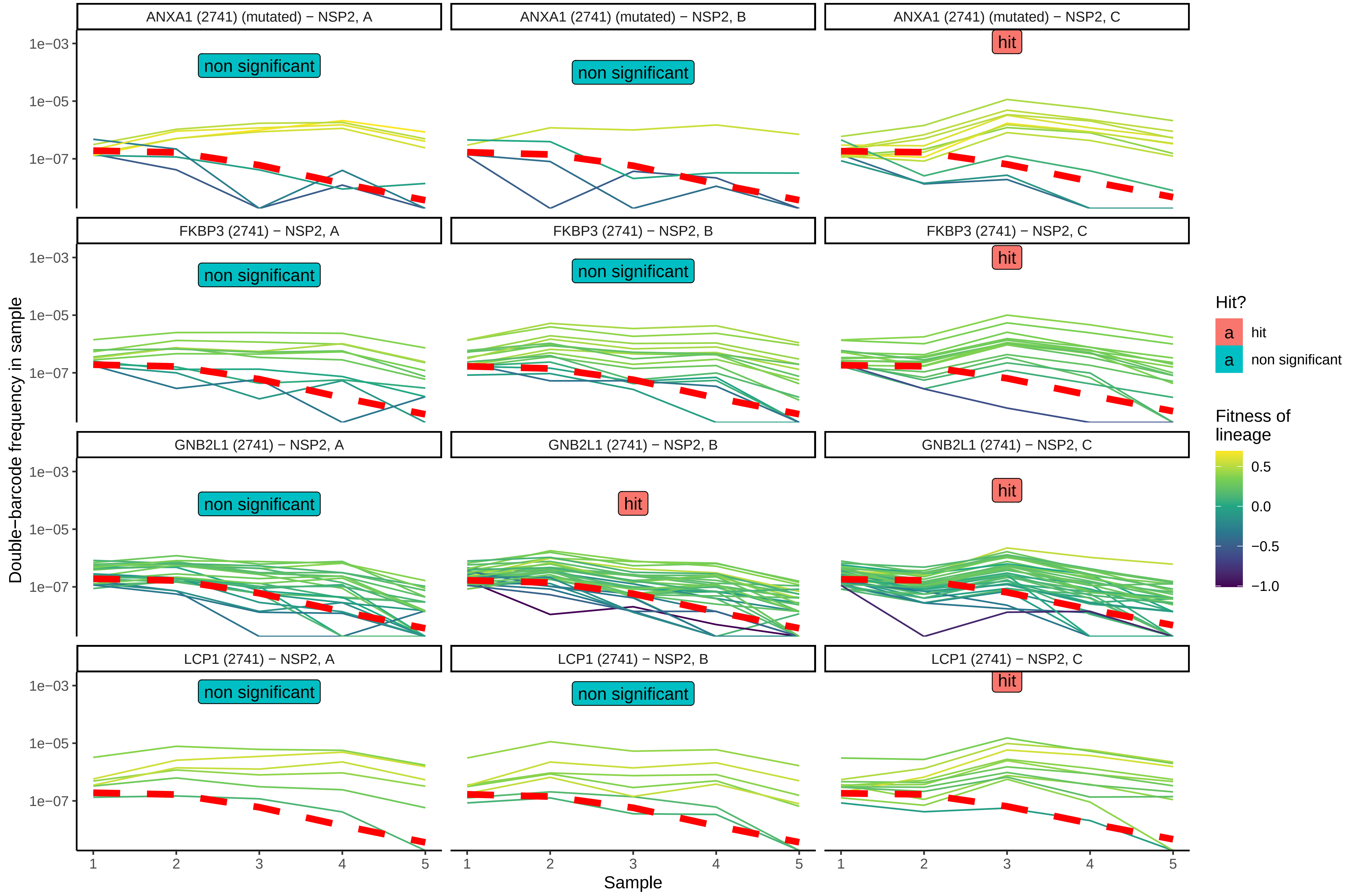

ANXA1 (2741) (mutated) − NSP2, A
ANXA1 (2741) (mutated) − NSP2, B
ANXA1 (2741) (mutated) − NSP2, C
FKBP3 (2741) − NSP2, A
FKBP3 (2741) − NSP2, B
FKBP3 (2741) − NSP2, C
GNB2L1 (2741) − NSP2, A
GNB2L1 (2741) − NSP2, B
GNB2L1 (2741) − NSP2, C
LCP1 (2741) − NSP2, A
LCP1 (2741) − NSP2, B
LCP1 (2741) − NSP2, C
non significant
hit
1e−03
1e−05
1e−07
Double−barcode frequency in sample
1
2
3
4
5
Sample
Hit?
a hit
a non significant
Fitness of lineage
0.5
0.0
−0.5
−1.0

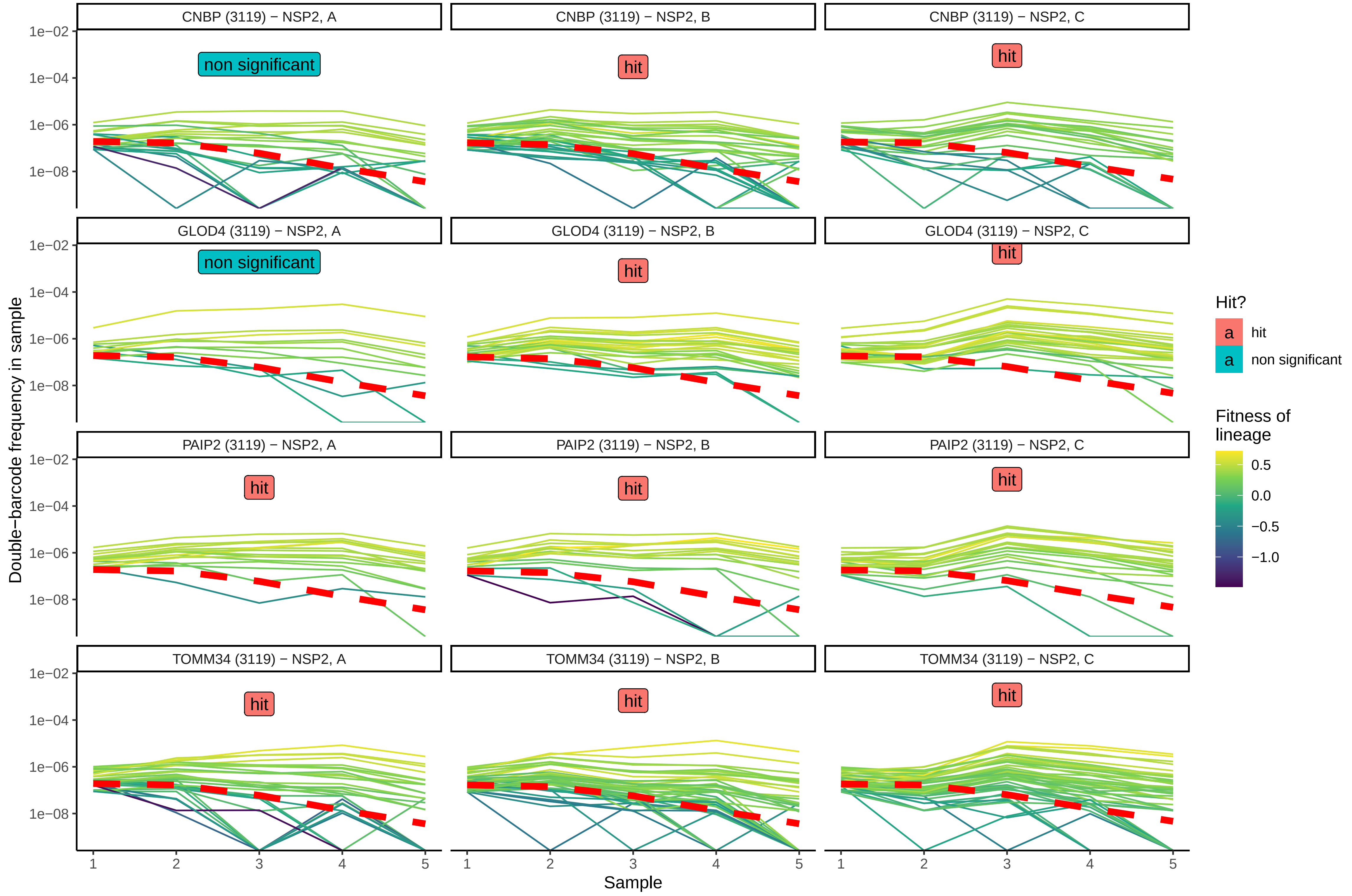

CNBP (3119) − NSP2, A
CNBP (3119) − NSP2, B
CNBP (3119) − NSP2, C
non significant
hit
hit
GLOD4 (3119) − NSP2, A
GLOD4 (3119) − NSP2, B
GLOD4 (3119) − NSP2, C
non significant
hit
hit
PAIP2 (3119) − NSP2, A
PAIP2 (3119) − NSP2, B
PAIP2 (3119) − NSP2, C
hit
hit
hit
TOMM34 (3119) − NSP2, A
TOMM34 (3119) − NSP2, B
TOMM34 (3119) − NSP2, C
hit
hit
hit
1e−02
1e−04
1e−06
1e−08
Double−barcode frequency in sample
1
2
3
4
5
Sample
Hit?
a hit
a non significant
Fitness of lineage
0.5
0.0
−0.5
−1.0

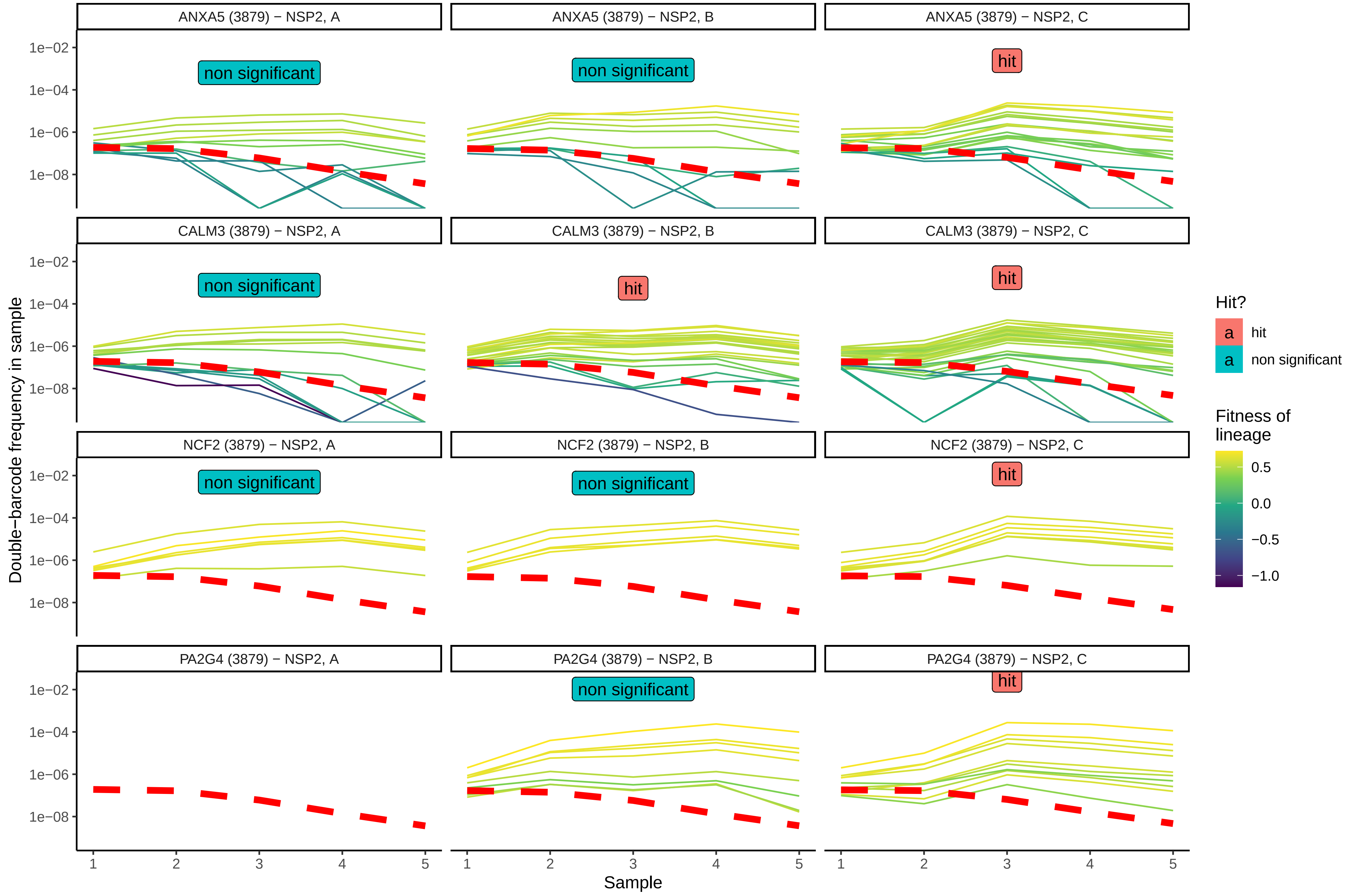

ANXA5 (3879) − NSP2, A
ANXA5 (3879) − NSP2, B
ANXA5 (3879) − NSP2, C
CALM3 (3879) − NSP2, A
CALM3 (3879) − NSP2, B
CALM3 (3879) − NSP2, C
NCF2 (3879) − NSP2, A
NCF2 (3879) − NSP2, B
NCF2 (3879) − NSP2, C
PA2G4 (3879) − NSP2, A
PA2G4 (3879) − NSP2, B
PA2G4 (3879) − NSP2, C
non significant
hit
Double−barcode frequency in sample
1e−02
1e−04
1e−06
1e−08
Sample
1
2
3
4
5
Hit?
a hit
a non significant
Fitness of lineage
0.5
0.0
−0.5
−1.0

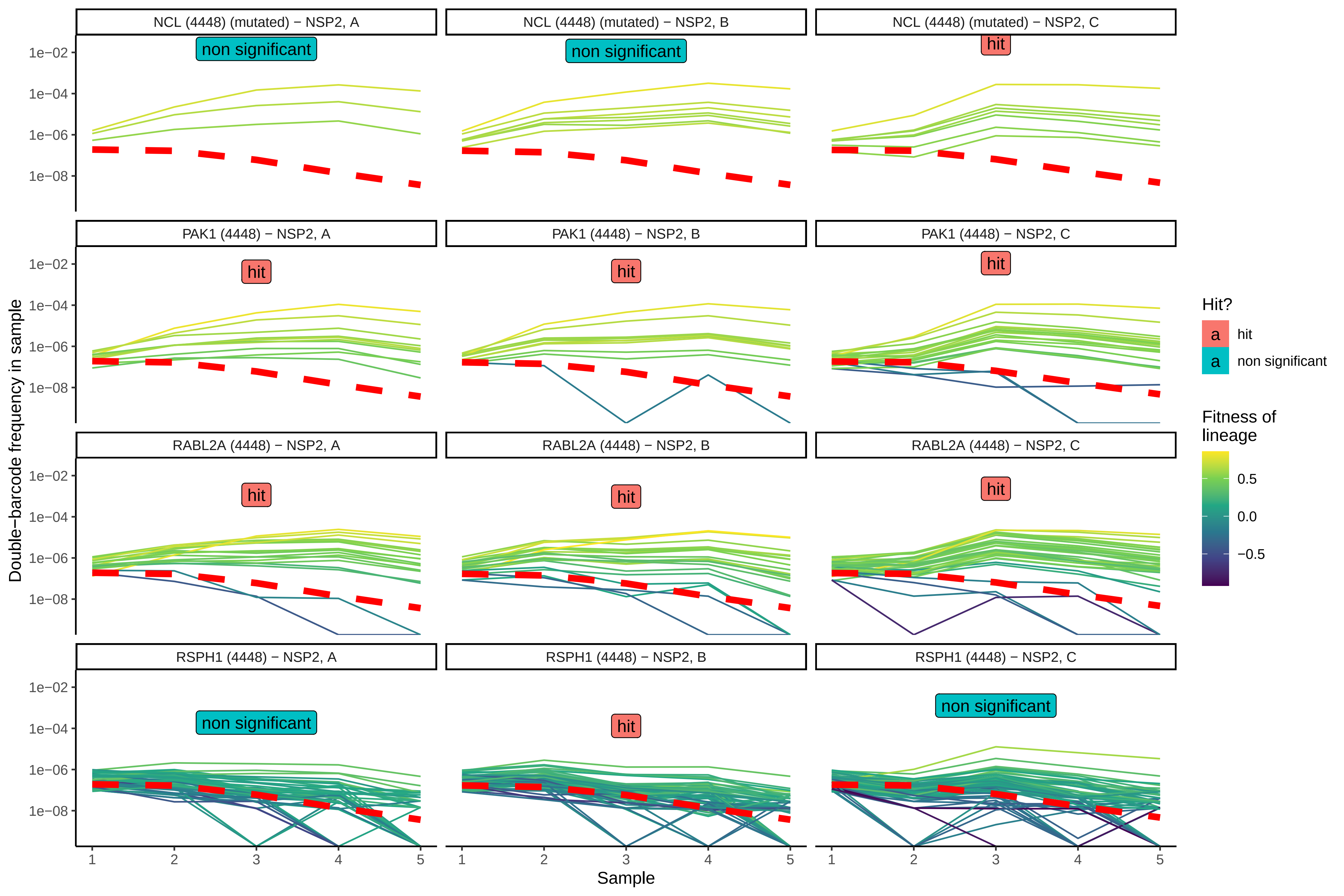

NCL (4448) (mutated) − NSP2, A
NCL (4448) (mutated) − NSP2, B
NCL (4448) (mutated) − NSP2, C
PAK1 (4448) − NSP2, A
PAK1 (4448) − NSP2, B
PAK1 (4448) − NSP2, C
RABL2A (4448) − NSP2, A
RABL2A (4448) − NSP2, B
RABL2A (4448) − NSP2, C
RSPH1 (4448) − NSP2, A
RSPH1 (4448) − NSP2, B
RSPH1 (4448) − NSP2, C
non significant
hit
1e−02
1e−04
1e−06
1e−08
Double−barcode frequency in sample
Sample
1
2
3
4
5
Hit?
hit
non significant
Fitness of lineage
0.5
0.0
−0.5

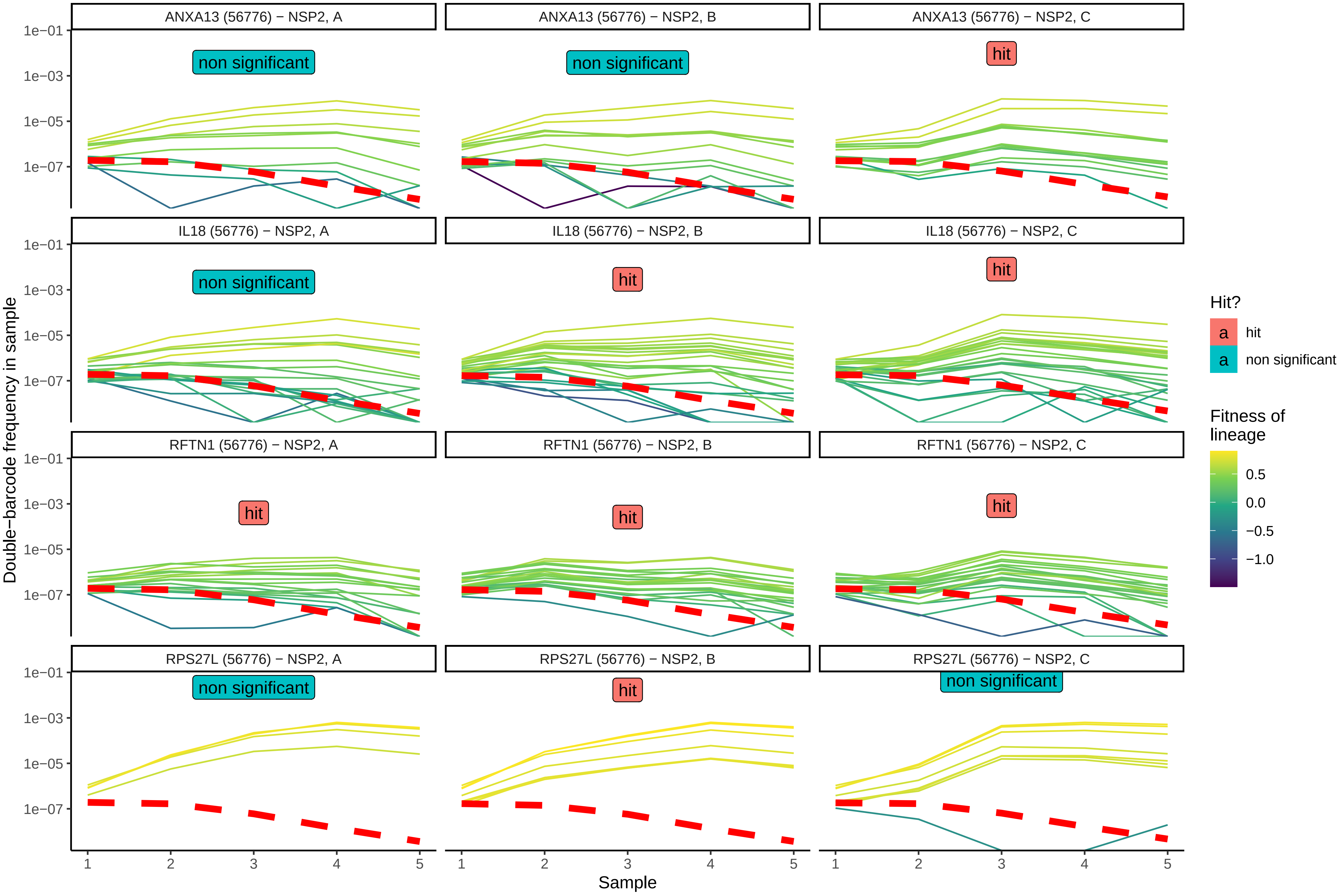

ANXA13 (56776) − NSP2, A
ANXA13 (56776) − NSP2, B
ANXA13 (56776) − NSP2, C
IL18 (56776) − NSP2, A
IL18 (56776) − NSP2, B
IL18 (56776) − NSP2, C
RFTN1 (56776) − NSP2, A
RFTN1 (56776) − NSP2, B
RFTN1 (56776) − NSP2, C
RPS27L (56776) − NSP2, A
RPS27L (56776) − NSP2, B
RPS27L (56776) − NSP2, C
non significant
hit
1e−01
1e−03
1e−05
1e−07
Double−barcode frequency in sample
Sample
1
2
3
4
5
Hit?
hit
non significant
Fitness of lineage
0.5
0.0
−0.5
−1.0

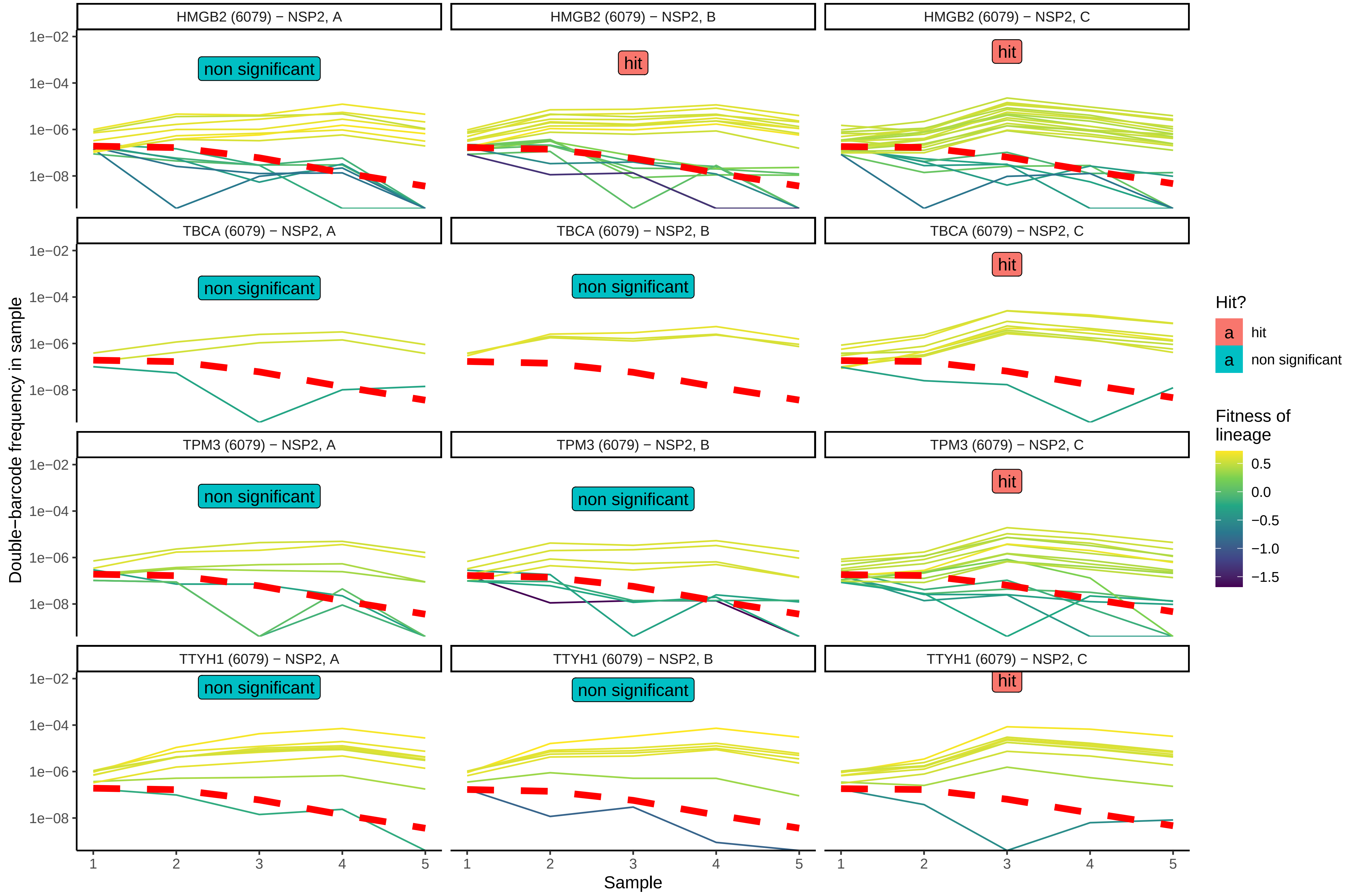

HMGB2 (6079) − NSP2, A
HMGB2 (6079) − NSP2, B
HMGB2 (6079) − NSP2, C
TBCA (6079) − NSP2, A
TBCA (6079) − NSP2, B
TBCA (6079) − NSP2, C
TPM3 (6079) − NSP2, A
TPM3 (6079) − NSP2, B
TPM3 (6079) − NSP2, C
TTYH1 (6079) − NSP2, A
TTYH1 (6079) − NSP2, B
TTYH1 (6079) − NSP2, C
non significant
hit
Double−barcode frequency in sample
1e−02
1e−04
1e−06
1e−08
Sample
1
2
3
4
5
Hit?
hit
non significant
Fitness of lineage
0.5
0.0
−0.5
−1.0
−1.5

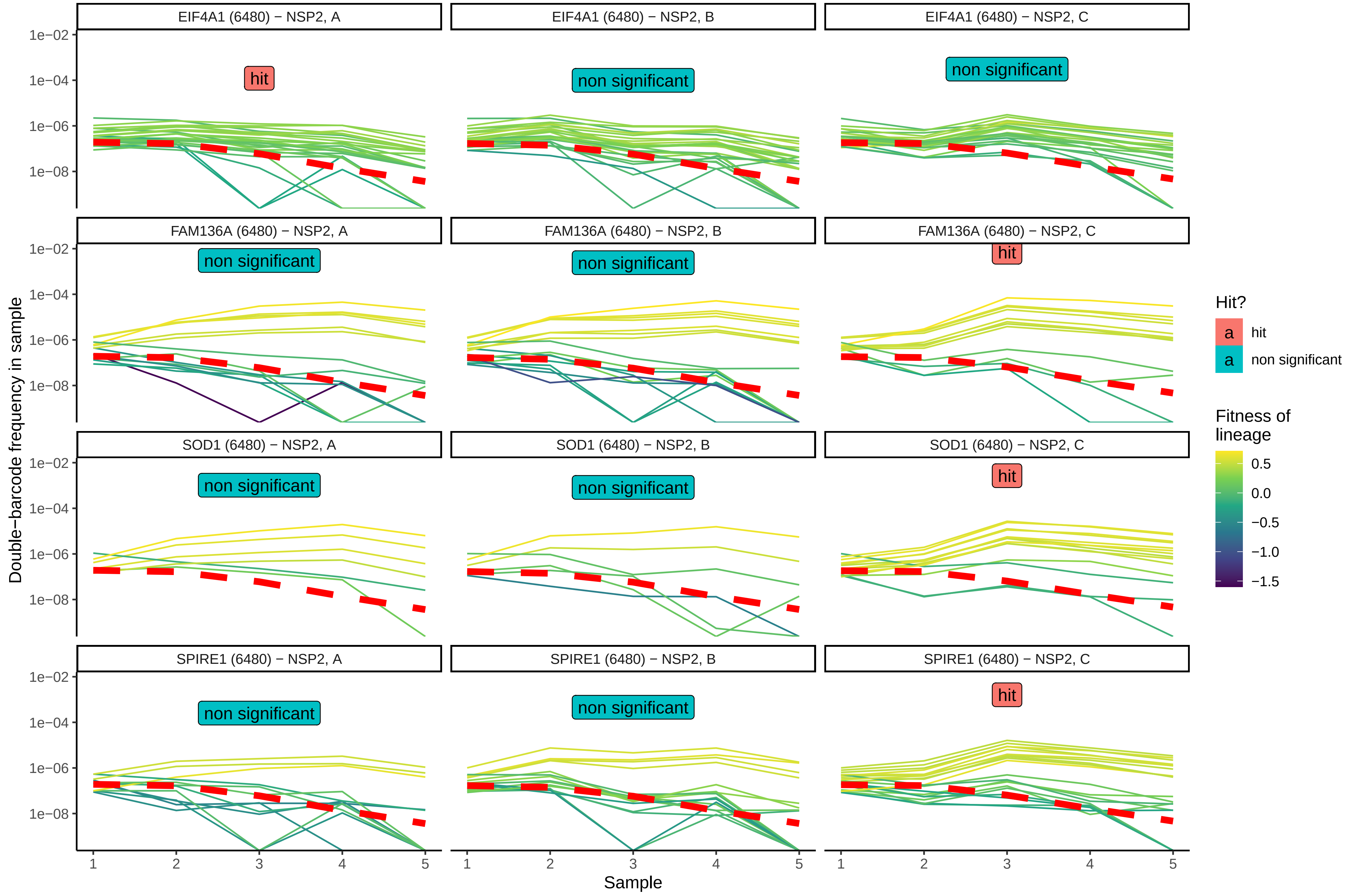

EIF4A1 (6480) − NSP2, A
EIF4A1 (6480) − NSP2, B
EIF4A1 (6480) − NSP2, C
hit
non significant
non significant
FAM136A (6480) − NSP2, A
FAM136A (6480) − NSP2, B
FAM136A (6480) − NSP2, C
non significant
non significant
hit
SOD1 (6480) − NSP2, A
SOD1 (6480) − NSP2, B
SOD1 (6480) − NSP2, C
non significant
non significant
hit
SPIRE1 (6480) − NSP2, A
SPIRE1 (6480) − NSP2, B
SPIRE1 (6480) − NSP2, C
non significant
non significant
hit
1e−02
1e−04
1e−06
1e−08
Double−barcode frequency in sample
1
2
3
4
5
Sample
Hit?
a hit
a non significant
Fitness of lineage
0.5
0.0
−0.5
−1.0
−1.5

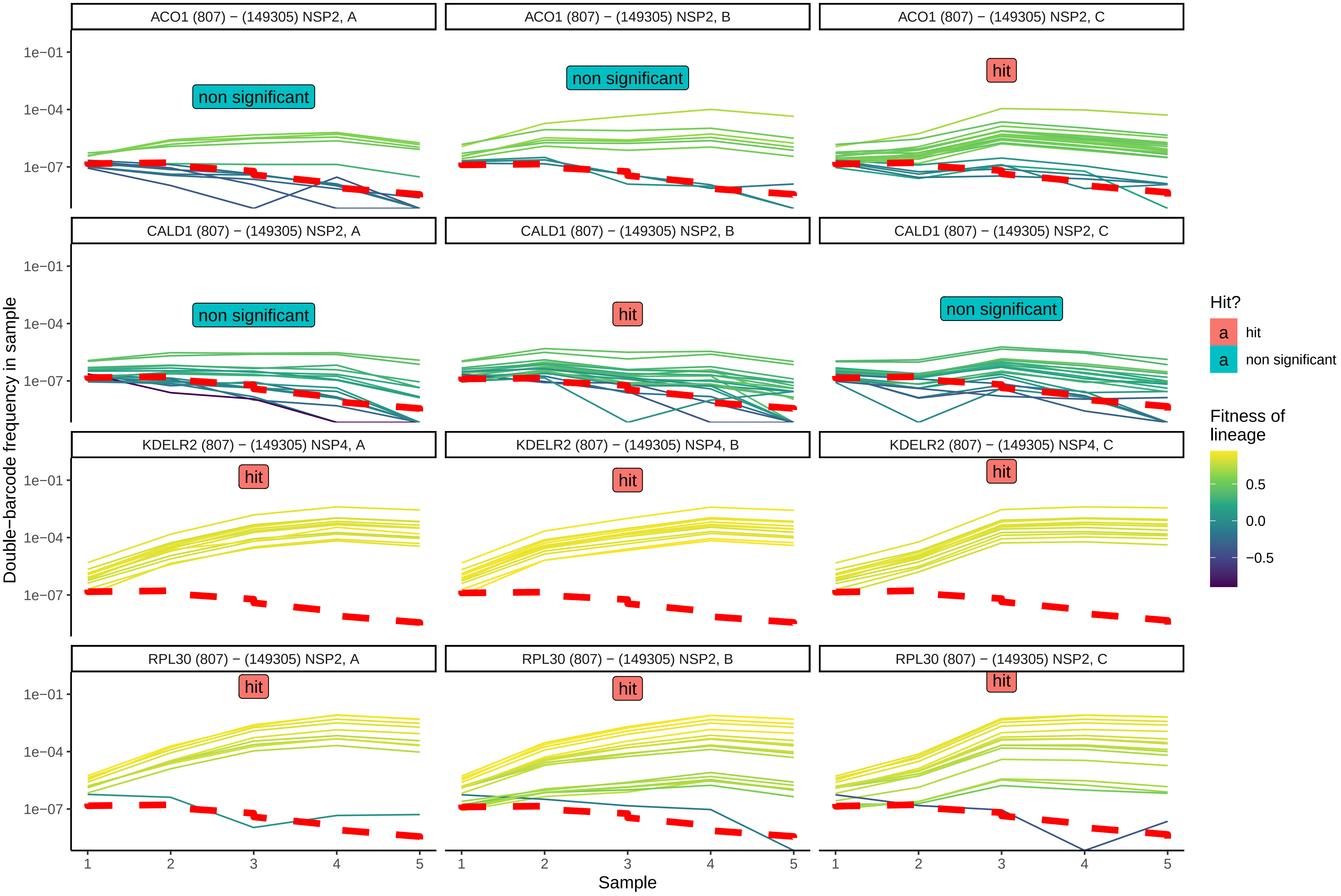

ACO1 (807) − (149305) NSP2, A
ACO1 (807) − (149305) NSP2, B
ACO1 (807) − (149305) NSP2, C
CALD1 (807) − (149305) NSP2, A
CALD1 (807) − (149305) NSP2, B
CALD1 (807) − (149305) NSP2, C
KDELR2 (807) − (149305) NSP4, A
KDELR2 (807) − (149305) NSP4, B
KDELR2 (807) − (149305) NSP4, C
RPL30 (807) − (149305) NSP2, A
RPL30 (807) − (149305) NSP2, B
RPL30 (807) − (149305) NSP2, C
non significant
hit
1e−01
1e−04
1e−07
1
2
3
4
5
Double−barcode frequency in sample
Sample
Hit?
hit
non significant
Fitness of lineage
0.5
0.0
−0.5

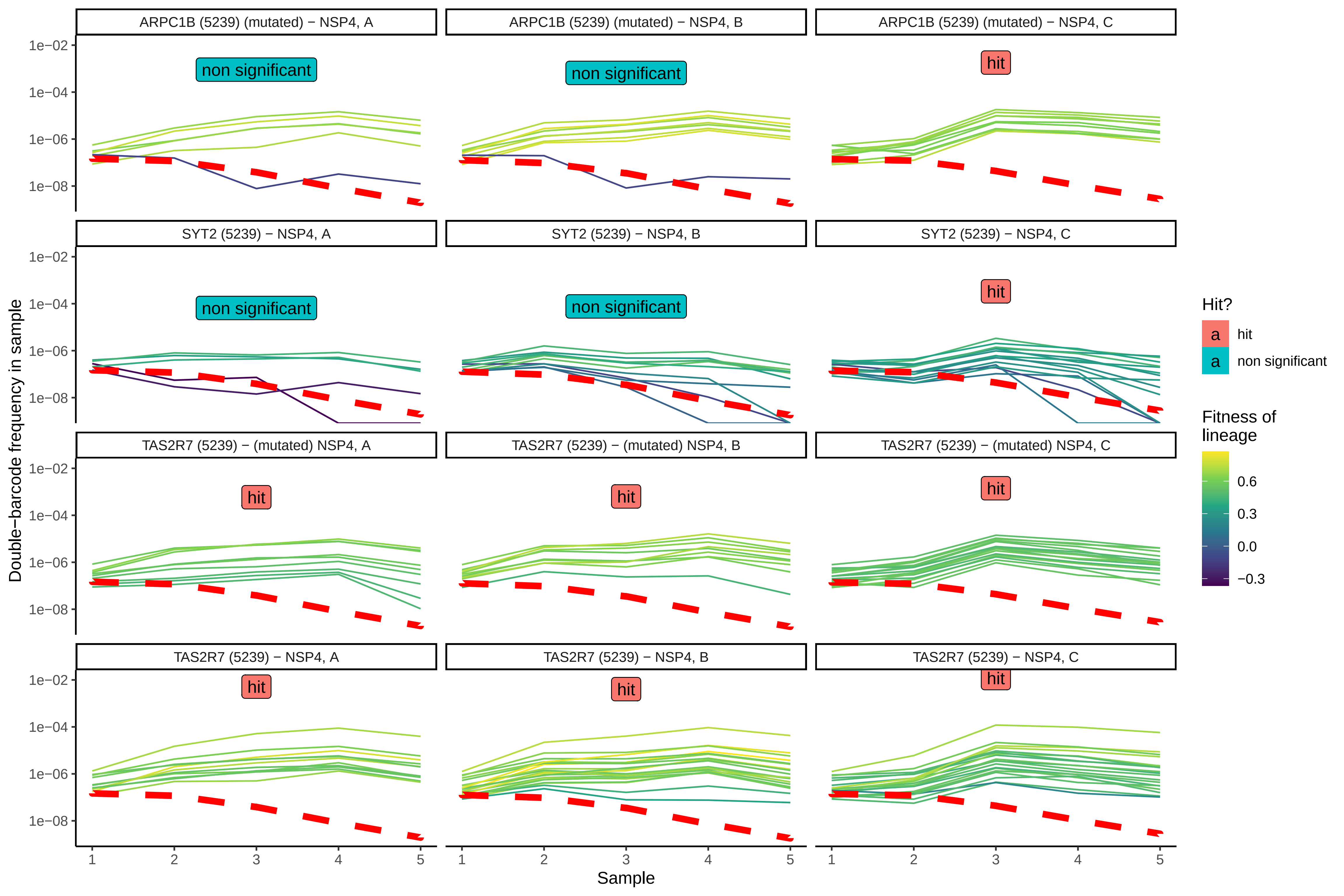

ARPC1B (5239) (mutated) − NSP4, A
ARPC1B (5239) (mutated) − NSP4, B
ARPC1B (5239) (mutated) − NSP4, C
SYT2 (5239) − NSP4, A
SYT2 (5239) − NSP4, B
SYT2 (5239) − NSP4, C
TAS2R7 (5239) − (mutated) NSP4, A
TAS2R7 (5239) − (mutated) NSP4, B
TAS2R7 (5239) − (mutated) NSP4, C
TAS2R7 (5239) − NSP4, A
TAS2R7 (5239) − NSP4, B
TAS2R7 (5239) − NSP4, C
non significant
hit
1e−02
1e−04
1e−06
1e−08
Double−barcode frequency in sample
1
2
3
4
5
Sample
Hit?
a hit
a non significant
Fitness of lineage
0.6
0.3
0.0
−0.3

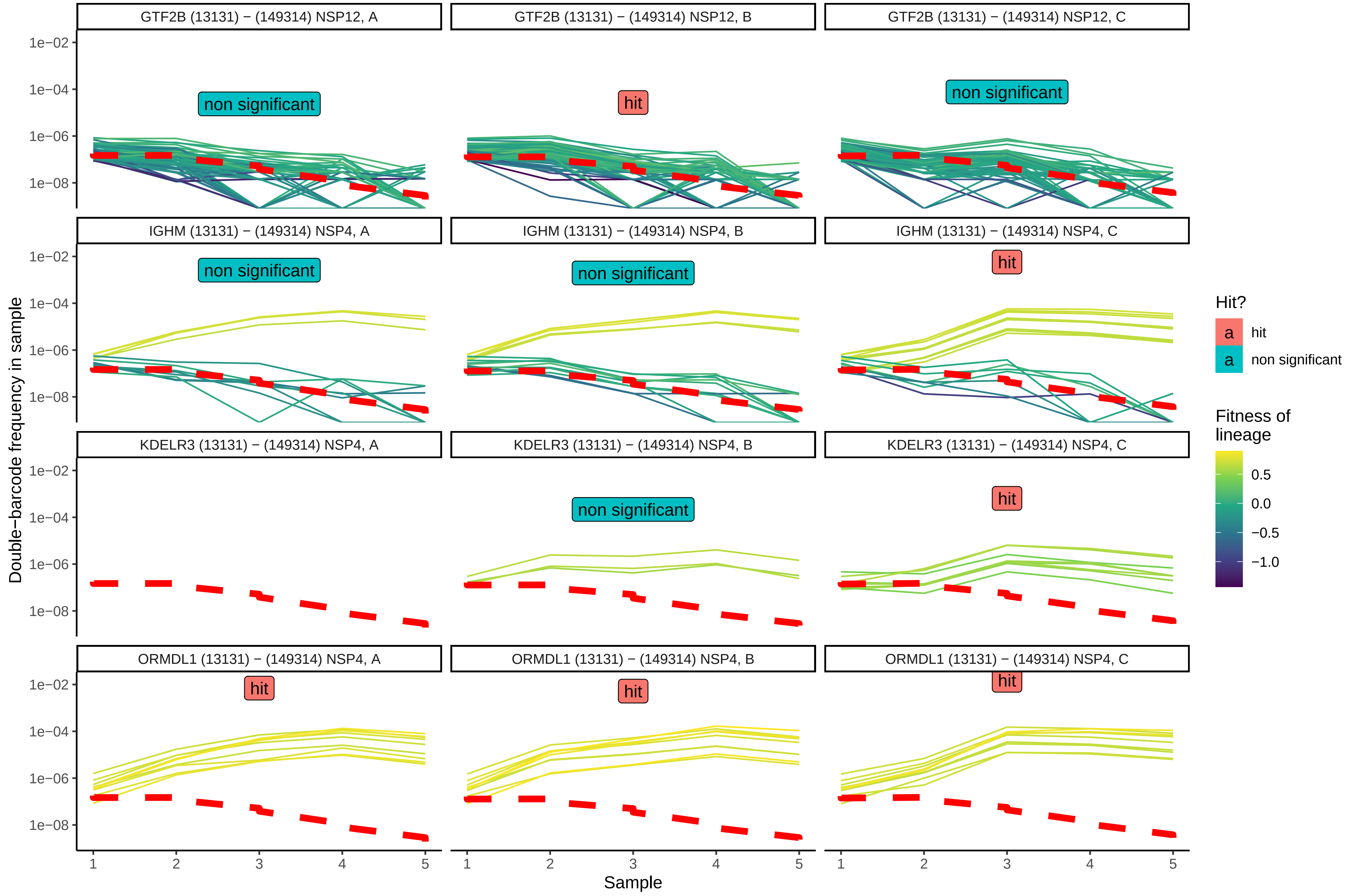
GTF2B (13131) − (149314) NSP12, A
GTF2B (13131) − (149314) NSP12, B
GTF2B (13131) − (149314) NSP12, C
non significant
hit
non significant
IGHM (13131) − (149314) NSP4, A
IGHM (13131) − (149314) NSP4, B
IGHM (13131) − (149314) NSP4, C
non significant
non significant
hit
KDELR3 (13131) − (149314) NSP4, A
KDELR3 (13131) − (149314) NSP4, B
KDELR3 (13131) − (149314) NSP4, C
non significant
hit
ORMDL1 (13131) − (149314) NSP4, A
ORMDL1 (13131) − (149314) NSP4, B
ORMDL1 (13131) − (149314) NSP4, C
hit
hit
hit
1e−02
1e−04
1e−06
1e−08
Double−barcode frequency in sample
1
2
3
4
5
Sample
Hit?
a hit
a non significant
Fitness of lineage
0.5
0.0
−0.5
−1.0

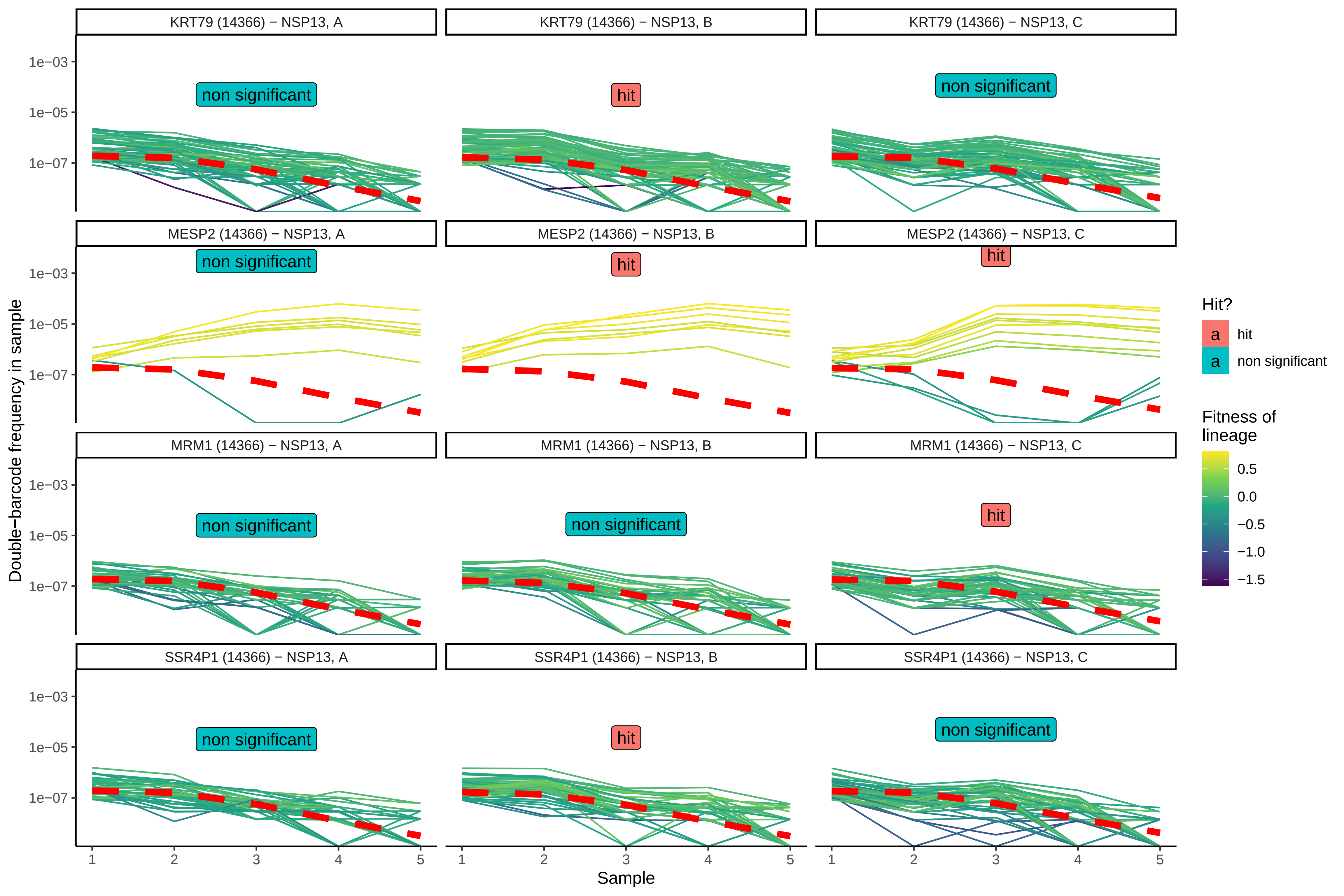

KRT79 (14366) − NSP13, A
KRT79 (14366) − NSP13, B
KRT79 (14366) − NSP13, C
MESP2 (14366) − NSP13, A
MESP2 (14366) − NSP13, B
MESP2 (14366) − NSP13, C
MRM1 (14366) − NSP13, A
MRM1 (14366) − NSP13, B
MRM1 (14366) − NSP13, C
SSR4P1 (14366) − NSP13, A
SSR4P1 (14366) − NSP13, B
SSR4P1 (14366) − NSP13, C
non significant
hit
1e−03
1e−05
1e−07
Double−barcode frequency in sample
Sample
1
2
3
4
5
Hit?
a hit
a non significant
Fitness of lineage
0.5
0.0
−0.5
−1.0
−1.5

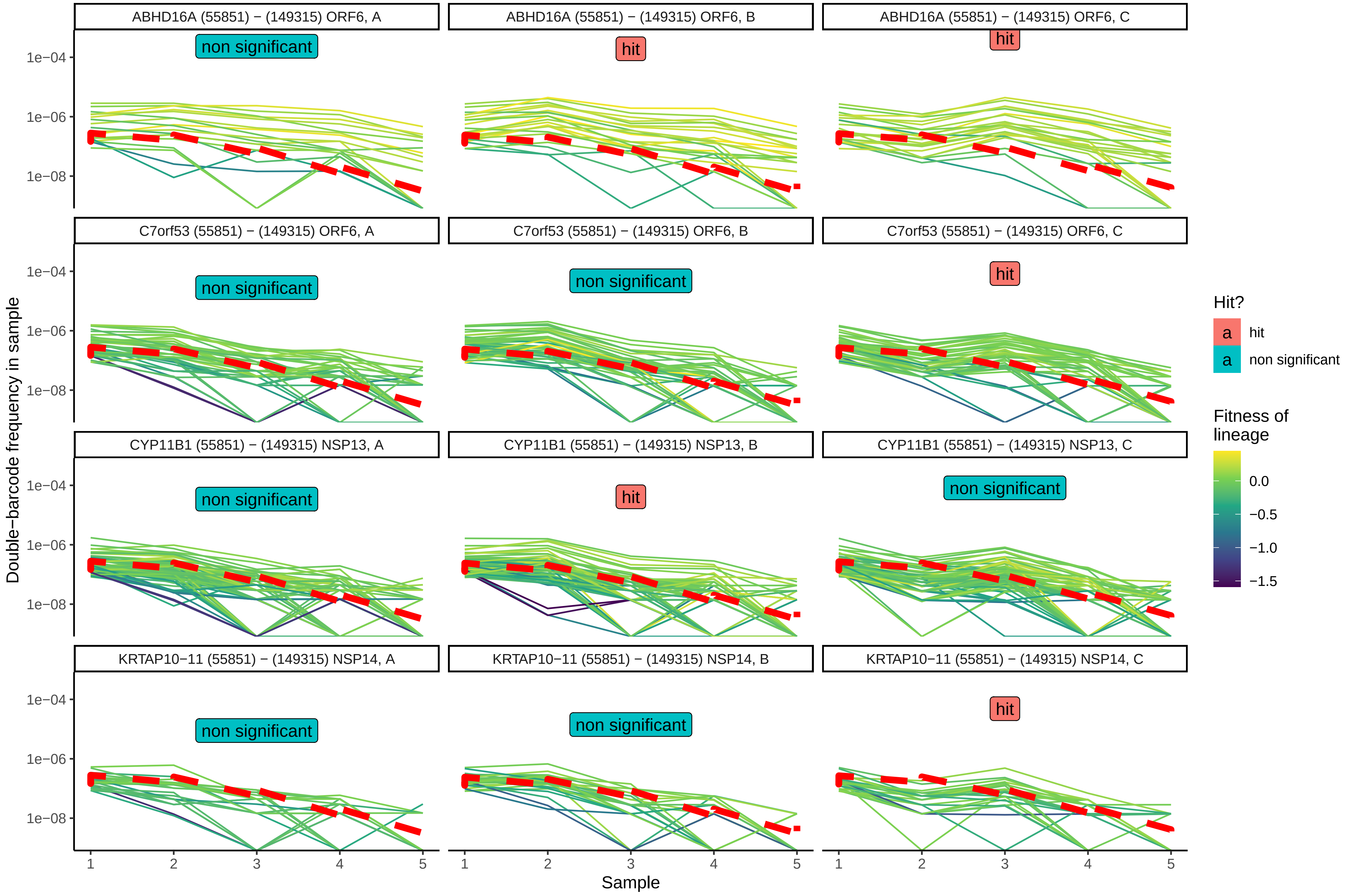

ABHD16A (55851) − (149315) ORF6, A
ABHD16A (55851) − (149315) ORF6, B
ABHD16A (55851) − (149315) ORF6, C
C7orf53 (55851) − (149315) ORF6, A
C7orf53 (55851) − (149315) ORF6, B
C7orf53 (55851) − (149315) ORF6, C
CYP11B1 (55851) − (149315) NSP13, A
CYP11B1 (55851) − (149315) NSP13, B
CYP11B1 (55851) − (149315) NSP13, C
KRTAP10−11 (55851) − (149315) NSP14, A
KRTAP10−11 (55851) − (149315) NSP14, B
KRTAP10−11 (55851) − (149315) NSP14, C
non significant
hit
Double−barcode frequency in sample
1e−04
1e−06
1e−08
Sample
1
2
3
4
5
Hit?
a hit
a non significant
Fitness of lineage
0.0
−0.5
−1.0
−1.5

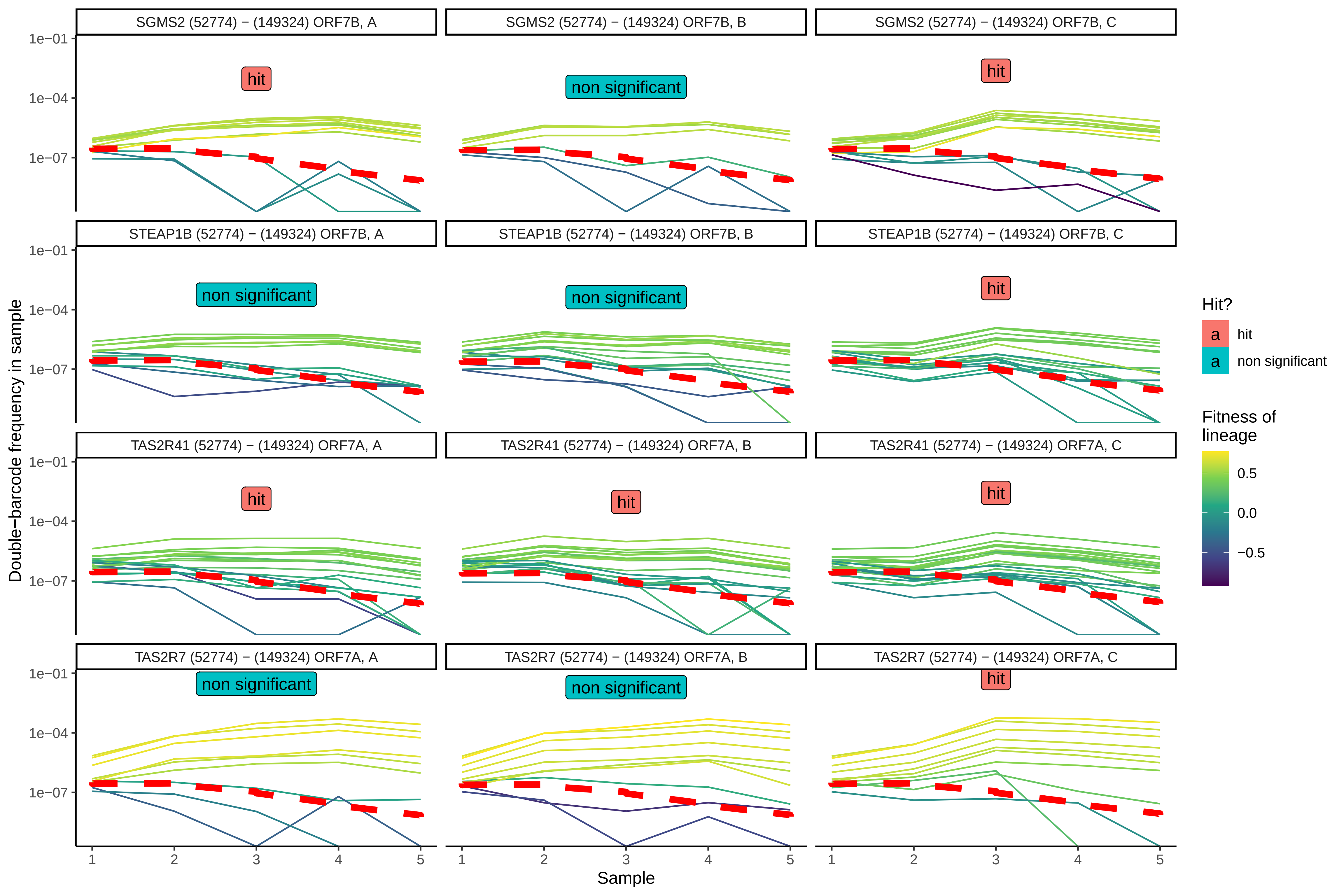
SGMS2 (52774) − (149324) ORF7B, A
SGMS2 (52774) − (149324) ORF7B, B
SGMS2 (52774) − (149324) ORF7B, C
STEAP1B (52774) − (149324) ORF7B, A
STEAP1B (52774) − (149324) ORF7B, B
STEAP1B (52774) − (149324) ORF7B, C
TAS2R41 (52774) − (149324) ORF7A, A
TAS2R41 (52774) − (149324) ORF7A, B
TAS2R41 (52774) − (149324) ORF7A, C
TAS2R7 (52774) − (149324) ORF7A, A
TAS2R7 (52774) − (149324) ORF7A, B
TAS2R7 (52774) − (149324) ORF7A, C
hit
non significant
hit
non significant
non significant
hit
hit
hit
hit
non significant
non significant
hit
1e−01
1e−04
1e−07
Double−barcode frequency in sample
Sample
1
2
3
4
5
Hit?
a hit
a non significant
Fitness of lineage
0.5
0.0
−0.5

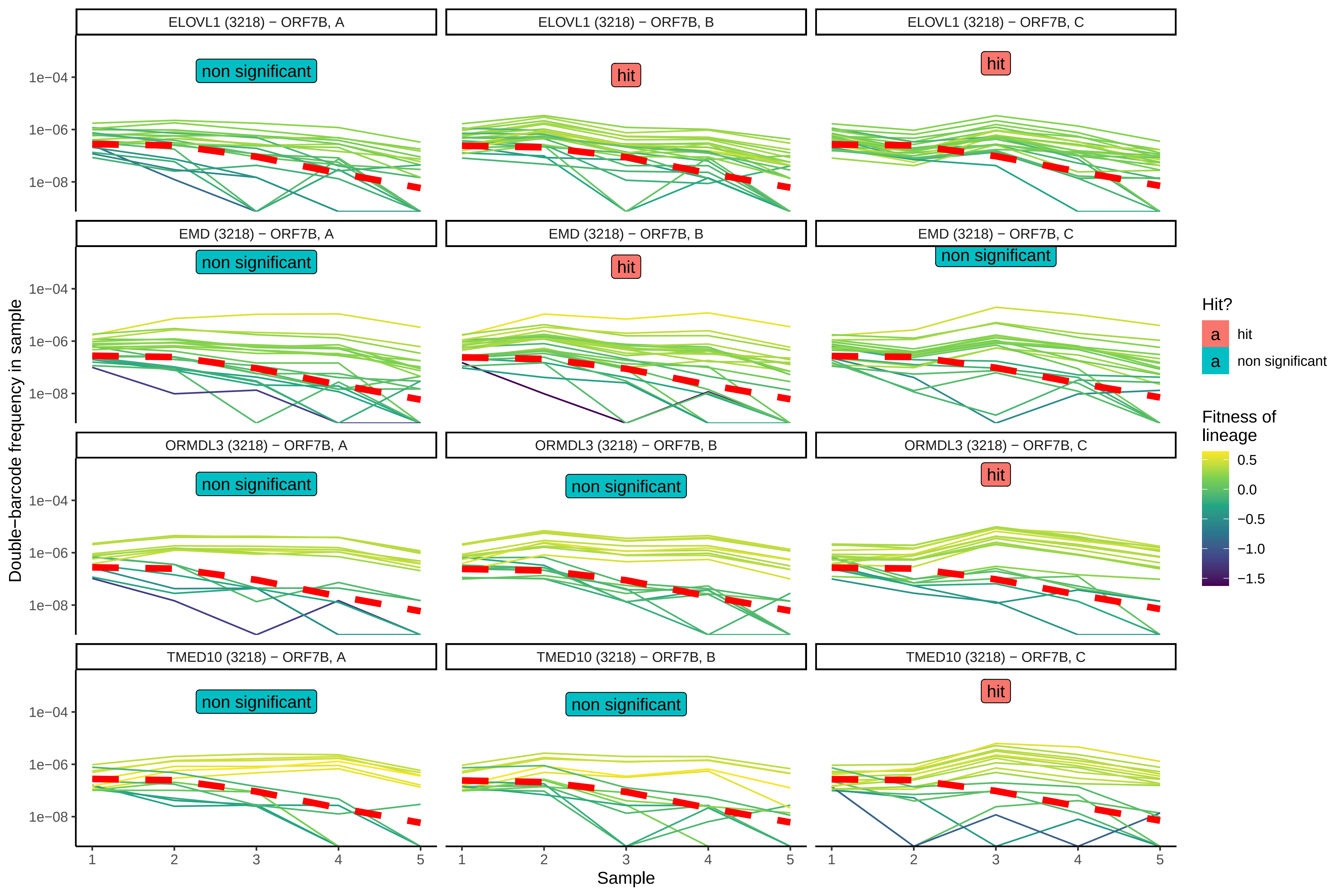

ELOVL1 (3218) − ORF7B, A
non significant
ELOVL1 (3218) − ORF7B, B
hit
ELOVL1 (3218) − ORF7B, C
hit
EMD (3218) − ORF7B, A
non significant
EMD (3218) − ORF7B, B
hit
EMD (3218) − ORF7B, C
non significant
ORMDL3 (3218) − ORF7B, A
non significant
ORMDL3 (3218) − ORF7B, B
non significant
ORMDL3 (3218) − ORF7B, C
hit
TMED10 (3218) − ORF7B, A
non significant
TMED10 (3218) − ORF7B, B
non significant
TMED10 (3218) − ORF7B, C
hit
1e−04
1e−06
1e−08
Double−barcode frequency in sample
1
2
3
4
5
Sample
Hit?
a hit
a non significant
Fitness of lineage
0.5
0.0
−0.5
−1.0
−1.5

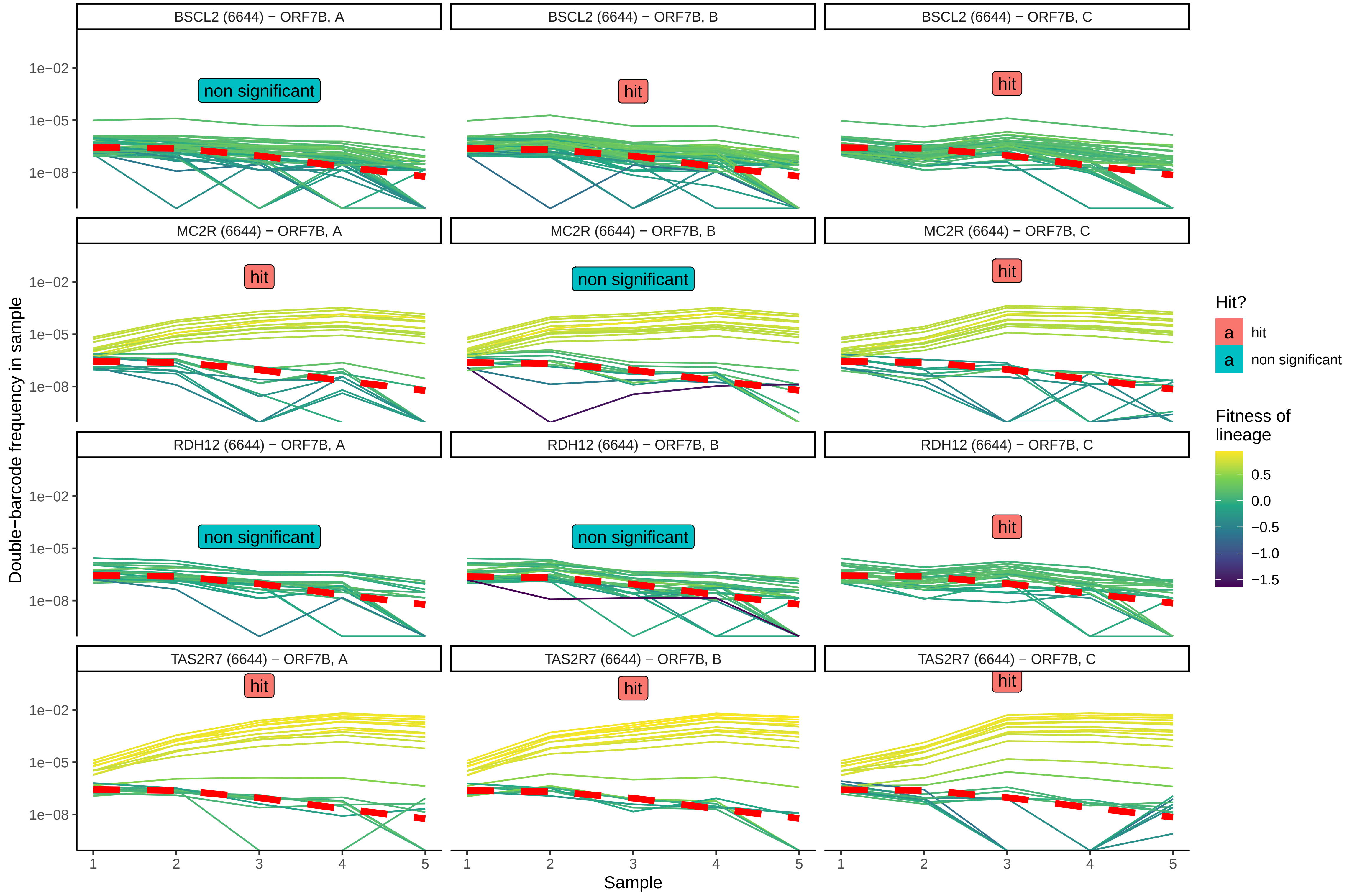

BSCL2 (6644) − ORF7B, A
BSCL2 (6644) − ORF7B, B
BSCL2 (6644) − ORF7B, C
MC2R (6644) − ORF7B, A
MC2R (6644) − ORF7B, B
MC2R (6644) − ORF7B, C
RDH12 (6644) − ORF7B, A
RDH12 (6644) − ORF7B, B
RDH12 (6644) − ORF7B, C
TAS2R7 (6644) − ORF7B, A
TAS2R7 (6644) − ORF7B, B
TAS2R7 (6644) − ORF7B, C
non significant
hit
1e−02
1e−05
1e−08
1
2
3
4
5
Sample
Double−barcode frequency in sample
Hit?
a hit
a non significant
Fitness of lineage
0.5
0.0
−0.5
−1.0
−1.5

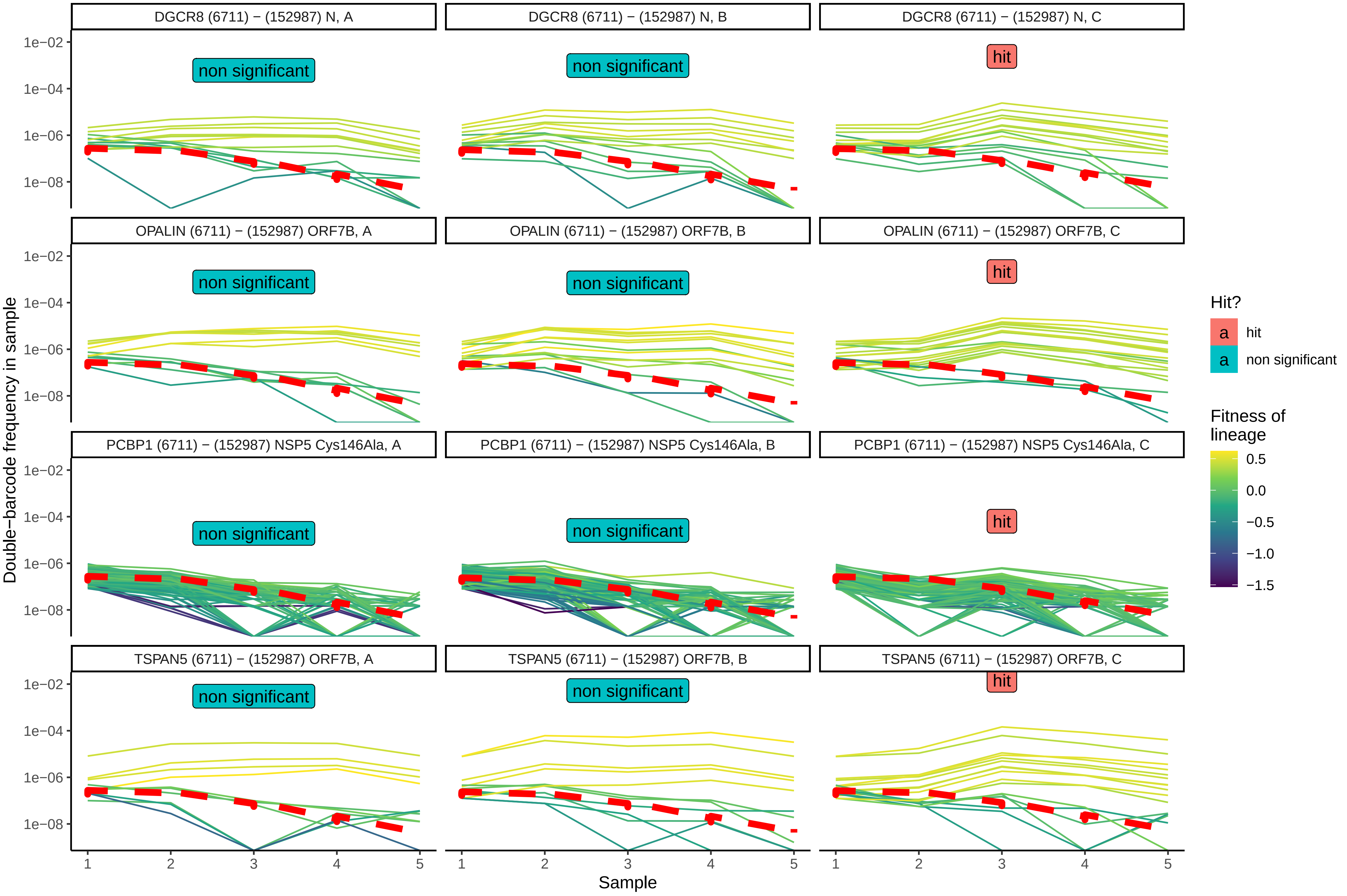

DGCR8 (6711) − (152987) N, A
DGCR8 (6711) − (152987) N, B
DGCR8 (6711) − (152987) N, C
OPALIN (6711) − (152987) ORF7B, A
OPALIN (6711) − (152987) ORF7B, B
OPALIN (6711) − (152987) ORF7B, C
PCBP1 (6711) − (152987) NSP5 Cys146Ala, A
PCBP1 (6711) − (152987) NSP5 Cys146Ala, B
PCBP1 (6711) − (152987) NSP5 Cys146Ala, C
TSPAN5 (6711) − (152987) ORF7B, A
TSPAN5 (6711) − (152987) ORF7B, B
TSPAN5 (6711) − (152987) ORF7B, C
non significant
hit
Double−barcode frequency in sample
1e−02
1e−04
1e−06
1e−08
Sample
1
2
3
4
5
Hit?
hit
non significant
Fitness of lineage
0.5
0.0
−0.5
−1.0
−1.5

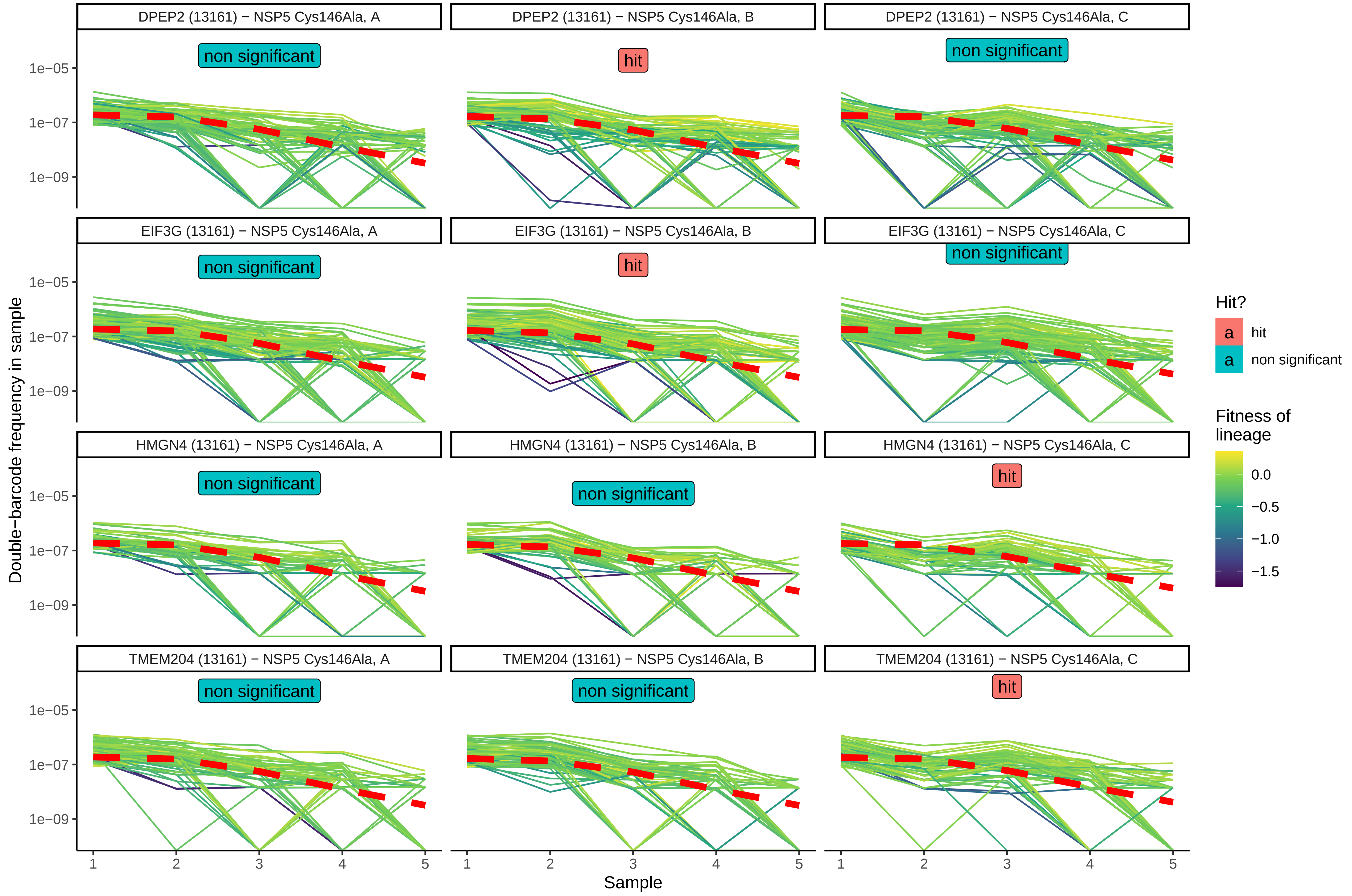

DPEP2 (13161) − NSP5 Cys146Ala, A
DPEP2 (13161) − NSP5 Cys146Ala, B
DPEP2 (13161) − NSP5 Cys146Ala, C
EIF3G (13161) − NSP5 Cys146Ala, A
EIF3G (13161) − NSP5 Cys146Ala, B
EIF3G (13161) − NSP5 Cys146Ala, C
HMGN4 (13161) − NSP5 Cys146Ala, A
HMGN4 (13161) − NSP5 Cys146Ala, B
HMGN4 (13161) − NSP5 Cys146Ala, C
TMEM204 (13161) − NSP5 Cys146Ala, A
TMEM204 (13161) − NSP5 Cys146Ala, B
TMEM204 (13161) − NSP5 Cys146Ala, C
non significant
hit
1e−05
1e−07
1e−09
Double−barcode frequency in sample
Sample
1
2
3
4
5
Hit?
a hit
a non significant
Fitness of lineage
0.0
−0.5
−1.0
−1.5

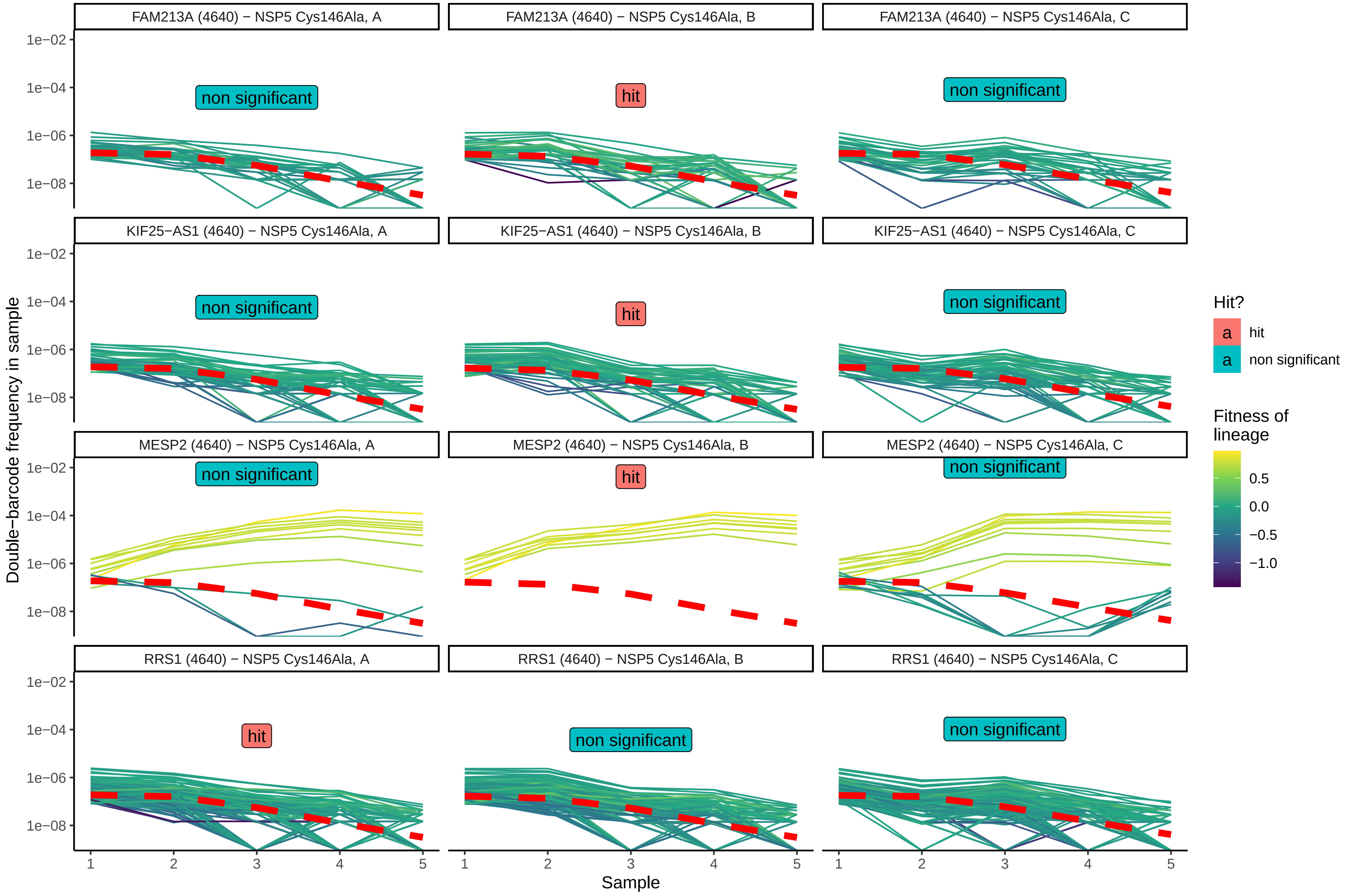
FAM213A (4640) − NSP5 Cys146Ala, A
FAM213A (4640) − NSP5 Cys146Ala, B
FAM213A (4640) − NSP5 Cys146Ala, C
KIF25−AS1 (4640) − NSP5 Cys146Ala, A
KIF25−AS1 (4640) − NSP5 Cys146Ala, B
KIF25−AS1 (4640) − NSP5 Cys146Ala, C
MESP2 (4640) − NSP5 Cys146Ala, A
MESP2 (4640) − NSP5 Cys146Ala, B
MESP2 (4640) − NSP5 Cys146Ala, C
RRS1 (4640) − NSP5 Cys146Ala, A
RRS1 (4640) − NSP5 Cys146Ala, B
RRS1 (4640) − NSP5 Cys146Ala, C
non significant
hit
Double−barcode frequency in sample
1e−02
1e−04
1e−06
1e−08
Sample
1
2
3
4
5
Hit?
hit
non significant
Fitness of lineage
0.5
0.0
−0.5
−1.0

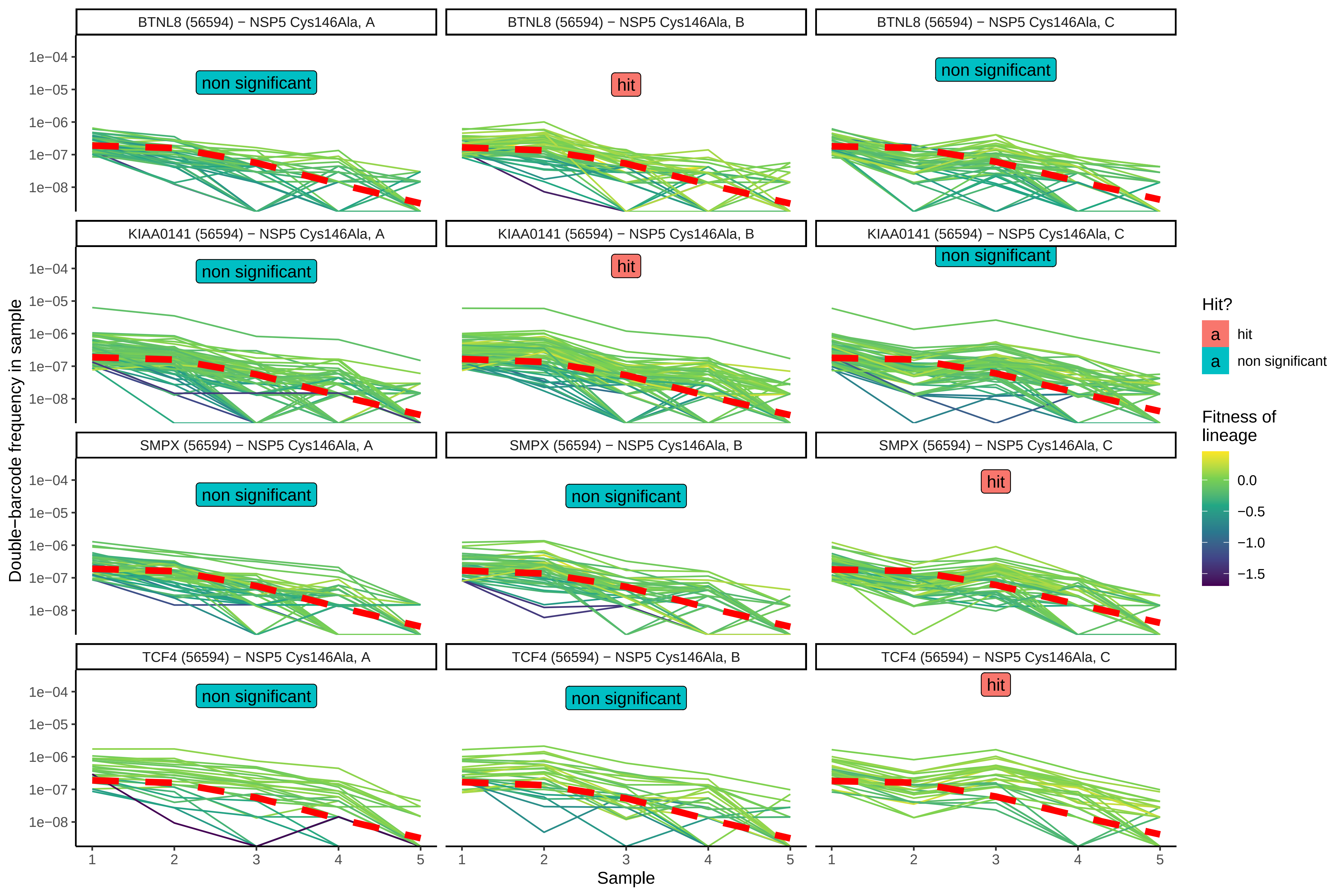
BTNL8 (56594) − NSP5 Cys146Ala, A
BTNL8 (56594) − NSP5 Cys146Ala, B
BTNL8 (56594) − NSP5 Cys146Ala, C
KIAA0141 (56594) − NSP5 Cys146Ala, A
KIAA0141 (56594) − NSP5 Cys146Ala, B
KIAA0141 (56594) − NSP5 Cys146Ala, C
SMPX (56594) − NSP5 Cys146Ala, A
SMPX (56594) − NSP5 Cys146Ala, B
SMPX (56594) − NSP5 Cys146Ala, C
TCF4 (56594) − NSP5 Cys146Ala, A
TCF4 (56594) − NSP5 Cys146Ala, B
TCF4 (56594) − NSP5 Cys146Ala, C
non significant
hit
1e−04
1e−05
1e−06
1e−07
1e−08
Double−barcode frequency in sample
1
2
3
4
5
Sample
Hit?
a hit
a non significant
Fitness of lineage
0.0
−0.5
−1.0
−1.5
