# Supplementary material for: Pooled PPIseq: Screening the SARS-CoV-2 and human interface with a scalable multiplexed protein-protein interaction assay platform
Source: PLoS One. 2025 Jan 17;20(1):e0299440. doi: 10.1371/journal.pone.0299440 (PMC11741623; doi:10.1371/journal.pone.0299440)
Supplement: S12 Fig — (PDF) [file pone.0299440.s018.pdf]

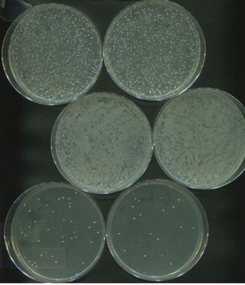

Yeast ySL508 was transformed on 2021-06-10 with a mixture of pRS426 (2micron plasmid, URA3) and PPIseq plasmids (lox5171, KanMX marker). The protocol used a Gietz-style PEG/LiAc and heatstress transformation, with a transient Cre-expression cassette and overnight YPGal induction, similar to the protocol included in supplemental.

In this image of YPD+G418 selection plates, the top row plates were recovered after post-heatstress centrifugation by resuspension in YPD, the middle resuspended in 1x Yeast Nitrogen Base + 2% glucose, the bottom in water. The left column was centrifuged at 800g after heatstress to remove the PEG/LiAc mixture, the right column was spun at 16,000g (max speed).

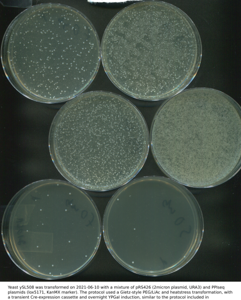

Yeast ySL508 was transformed on 2021-06-10 with a mixture of pRS426 (2micron plasmid, URA3) and PPIseq plasmids (lox5171, KanMX marker). The protocol used a Gietz-style PEG/LiAc and heatstress transformation, with a transient Cre-expression cassette and overnight YPGal induction, similar to the protocol included in supplemental.

In this image of YPD+G418 selection plates, the top row of plates were recovered after post-heatstress centrifugation by resuspension in YPD, the middle resuspended in 1x Yeast Nitrogen Base + 2% glucose, the bottom in water. The left column had the transformation mix prepared with sheared salmon sperm DNA from Thermo, the right column was with transformation mix prepared from unsheared salmon sperm DNA dissolved into solution carefully by Kevin Roy.

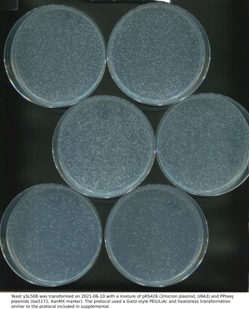

Yeast ySL508 was transformed on 2021-06-10 with a mixture of pRS426 (2micron plasmid, URA3) and PPIseq plasmids (lox5171, KanMX marker). The protocol used a Gietz-style PEG/LiAc and heatstress transformation similar to the protocol included in supplemental.

In this image, the top row of SC-URA selection plates were recovered after post-heatstress centrifugation by resuspension in YPD, the middle resuspended in 1x Yeast Nitrogen Base + 2% glucose, the bottom in water. The left column was centrifuged at 800g after heatstress to remove the PEG/LiAc mixture, the right column was spun at 16,000g (max speed).

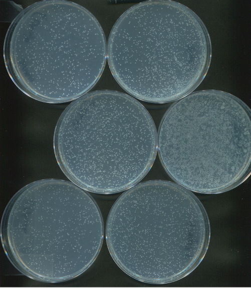

Yeast ySL508 was transformed on 2021-06-10 with a mixture of pRS426 (2micron plasmid, URA3) and PPIseq plasmids (lox5171, KanMX marker). The protocol used a Gietz-style PEG/LiAc and heatstress transformation similar to the protocol included in supplemental.

In this image of SC-URA selection plates, the top row of plates were recovered after post-heatstress centrifugation by resuspension in YPD, the middle resuspended in 1x Yeast Nitrogen Base + 2% glucose, the bottom in water. The left column had the transformation mix prepared with sheared salmon sperm DNA from Thermo, the right column was with transformation mix prepared from unsheared salmon sperm DNA dissolved into solution carefully by Kevin Roy.
