# Supplementary material for: Pooled PPIseq: Screening the SARS-CoV-2 and human interface with a scalable multiplexed protein-protein interaction assay platform
Source: PLoS One. 2025 Jan 17;20(1):e0299440. doi: 10.1371/journal.pone.0299440 (PMC11741623; doi:10.1371/journal.pone.0299440)
Supplement: Attachment — Submitted filename: PLoS One Review Miller et al Mar 2024.pdf [file pone.0299440.s038.pdf]

The manuscript by Miller et al. presents the application of a sequencing-based method for identifying protein-protein interactions (PPiSeq), a technique originally pioneered by the Levy lab in 2017. This study leverages PPiSeq to delineate viral-host protein-protein interactions (PPIs), a research area of significant interest that is in need of innovative scalable methodologies. By determining the host and viral partner proteins, this type of work can offer valuable insights into viral replication processes and facilitate the development of novel antiviral therapies. The experimental design and analyses conducted are robust, rendering the manuscript suitable for publication in PLoS One. While no major revisions are required, a few minor adjustments are recommended prior to acceptance.

Major Revisions:
None

Minor Revisions:
1. Line 130: Correct “lading” to “landing”

2. The authors conduct a statistical analysis to demonstrate that their method significantly overlaps with datasets previously published by IMex. However, despite the statistical validation, the overall number of overlaps remains small. It would be beneficial for the authors to delve deeper into some of the novel protein-protein interactions (PPIs) that have not been identified before. The authors should provide some discussion of why this approach might have picked up interactions the previous approaches missed. On the other hand, although more challenging, it would also be insightful to investigate which PPIs, potentially replicated by different laboratories independently, do not appear among the 74 hits identified through PPiSeq. Expanding the discussion to address these aspects would greatly enrich the manuscript, offering a more comprehensive understanding of the method's significance and the novelty of the findings.

3. Within the 113 top hits (FDR<0.05) we see NSP2 52 times, ORF7 22 times, NSP4 16 times, NSP5C145A 13 times – can the authors determine if there is any relationship between the expression levels of these viral genes and observing interactions?
