# Supplementary material for: Pooled PPIseq: Screening the SARS-CoV-2 and human interface with a scalable multiplexed protein-protein interaction assay platform
Source: PLoS One. 2025 Jan 17;20(1):e0299440. doi: 10.1371/journal.pone.0299440 (PMC11741623; doi:10.1371/journal.pone.0299440)
Supplement: Attachment — Submitted filename: PONE-D-24-05663.pdf [file pone.0299440.s039.pdf]

21 May 2024

The Editor

PLOS ONE
plosone@plos.org

Manuscript Number: PONE-D-24-05663

Manuscript Title: Pooled PPIseq: screening the SARS-CoV-2 and human interface with a scalable multiplexed protein-protein interaction assay platform

My assessment / comments are as follows:

1. Is the manuscript technically sound, and do the data support the conclusions?
   Yes

2. Has the statistical analysis been performed appropriately and rigorously?
   Yes
   I noticed that the format for reporting p values differ throughout the manuscript (p value < 1e5 [line 329], p value < 0.05 [line 357], p value < 1e-6 [line 337], etc.). Is there a reason?

3. Have the authors made all data underlying the findings in their manuscript fully available?
   Yes

4. Is the manuscript presented in an intelligible fashion and written in standard English?
   Yes

5. Review comments to the Author:
   Mostly editorial apart from above comments.

   a. Pag 7 – inconsistent use of bold format

   b. Page 10 – starting a sentence with a number (line 264) makes reading difficult. The sentence needs to be reconstructed or 211,688 should be written out in full.

   c. Page 14 line 337 – seems like a misplaced)

   d. Page 16 line 410 – no space between "…s[81]"

   e. Methods – unless this is customary for this specific journal, the degree symbol must be used for temperatures (72°C instead of 72C)

Stellenbosch UNIVERSITY IYUNIVESITHI UNIVERSITEIT
Fakulteit Geneeskunde en Gesondheidswetenskappe
Faculty of Medicine and Health Sciences
Prof Corena de Beer,
Chair: Postgraduate Program Committee / Senior Lecturer / Principal Medical Scientist
Division of Medical Virology, Department of Pathology, Clinical Building, Faculty of Medicine and Health Sciences,
Stellenbosch University, P O Box 241, CAPE TOWN, 7505, South Africa
Tel: +27 27 938 9453, 082 826 0453 / Fax: +27 21 938 9361, 086 621 3268 / e-mail: cdeb@sun.ac.za
NATIONAL HEALTH LABORATORY SERVICE

f. The figures are quite complex and difficult to follow. For example, Figure 1 consists of 10 sections – can this not be split into more, simpler and easier to follow figures?

g. Minor spelling mistakes.

I found this manuscript of exceptionally high quality and although it is fairly lengthy, I am of the opinion that the information contained therein, justifies the length.

Kind Regards

Prof Corena de Beer | PhD

Associate Professor | Principal Medical Scientist

Chair: Stellenbosch University, Post Graduate Programme Committee (Medical Virology);
Vice-chair: Research Ethics Committee - Biological and Environmental Safety
Chair: National Department of Health, National Task Force (WHO Global Polio Eradication)

Faculty of Medicine and Health Sciences | National Health Laboratory Service
Room 8002, Clinical Building, Francie van Zijl Drive, Tygerberg Campus | South Africa

Tel: +27 21 938 9453 | +27 82 826 0453; Fax: +27 21 938 9361 | 086 621 3268
cdeb@sun.ac.za | corena.debeer@nhls.ac.za
www.sun.ac.za | www.sun.ac.za/english/faculty/healthsciences/virology/Pages/Medical_Virology.aspx

Stellenbosch UNIVERSITY IYUNIVESITHI UNIVERSITEIT   NATIONAL HEALTH LABORATORY SERVICE
